# Single PbS colloidal quantum dot transistors

**DOI:** 10.1038/s41467-023-43343-7

**Published:** 2023-11-18

**Authors:** Kenji Shibata, Masaki Yoshida, Kazuhiko Hirakawa, Tomohiro Otsuka, Satria Zulkarnaen Bisri, Yoshihiro Iwasa

**Affiliations:** 1 Department of Electrical and Electronic Engineering, Tohoku Institute of Technology, 35-1 Yagiyama, Kasumi-cho, Taihaku-ku, Sendai, 982-8577 Japan; 2 Institute of Industrial Science, University of Tokyo, 4-6-1 Komaba, Meguro-ku, Tokyo, 153-8505 Japan; 3 Institute for Nano Quantum Information Electronics, University of Tokyo, 4-6-1 Komaba, Meguro-ku, Tokyo, 153-8505 Japan; 4 Research Institute of Electrical Communication, Tohoku University, 2-1-1 Katahira, Aoba-ku, Sendai, 980-8577 Japan; 5 WPI Advanced Institute for Materials Research, Tohoku University, Sendai, 980-8577 Japan; 6 Department of Electronic Engineering, Tohoku University, Aoba 6-6-05, Aramaki, Aoba-Ku, Sendai, 980-8579 Japan; 7 Center for Science and Innovation in Spintronics, Tohoku University, 2-1-1 Katahira, Aoba-ku, Sendai, 980-8577 Japan; 8 Quantum Functional System Research Group, RIKEN Center for Emergent Matter Science, 2-1 Hirosawa, Wako, Saitama, 351-0198 Japan; 9 Emergent Device Research Team, RIKEN Center for Emergent Matter Science, 2-1 Hirosawa Wako, Saitama, 351-0198 Japan; 10 Department of Applied Physics and Chemical Engineering, Tokyo University of Agriculture and Technology, 2-24-16 Naka-cho, Koganei-shi, Tokyo, 184-8588 Japan; 11 Department of Applied Physics and Quantum-Phase Electronics Center, University of Tokyo, Hongo, Bunkyo-ku, Tokyo, 113-8656 Japan

**Keywords:** Electronic devices, Quantum dots, Quantum dots

## Abstract

Colloidal quantum dots are sub-10 nm semiconductors treated with liquid processes, rendering them attractive candidates for single-electron transistors operating at high temperatures. However, there have been few reports on single-electron transistors using colloidal quantum dots due to the difficulty in fabrication. In this work, we fabricated single-electron transistors using single oleic acid-capped PbS quantum dot coupled to nanogap metal electrodes and measured single-electron tunneling. We observed dot size-dependent carrier transport, orbital-dependent electron charging energy and conductance, electric field modulation of the electron confinement potential, and the Kondo effect, which provide nanoscopic insights into carrier transport through single colloidal quantum dots. Moreover, the large charging energy in small quantum dots enables single-electron transistor operation even at room temperature. These findings, as well as the commercial availability and high stability, make PbS quantum dots promising for the development of quantum information and optoelectronic devices, particularly room-temperature single-electron transistors with excellent optical properties.

## Abstract (web summary)

Colloidal quantum dots remain unexplored for applications in single-electron devices. Here, the authors demonstrate single-electron transistors using single PbS colloidal quantum dot, highlighting their room-temperature operation.

## Introduction

Colloidal quantum dots (CQDs) are small semiconductor crystals with a diameter of several nanometers^1^. CQDs exhibit excellent light emission/absorption characteristics, and their optical bandgaps are widely tunable by adjusting their size. CQDs can be treated by liquid processes, and their functionality can be controlled by selecting suitable ligands. These properties make CQDs attractive candidates for use as transport channels in single-electron transistors (SETs) operating at high temperatures. However, there have been only a few evaluations of carrier transport through single CQDs in transistor geometries, which are limited to CdSe^2^, PbS^3^, HgSe^4^, and CdSe core-shell CQD systems^5^ measured only at cryogenic temperatures because of the technical difficulties in electrically accessing single CQDs prepared by bottom-up methods. To date, SETs using semiconductor quantum dots have mainly been fabricated in GaAs^6–9^, InAs^10–13^, and Si-based quantum dots^14–18^. These quantum dots are typically ~50–100 nm in size, leading to a small Coulomb charging energy of <~10 meV. Therefore, most SETs operate only at cryogenic temperatures, which limits their practical applications. Through the utilization of the small size of CQDs, high-temperature SET operation is expected in single CQD transistors, which may serve as a basis for many single-electron devices^19^.

Moreover, improved electrical manipulation and read out of quantum states in single CQDs allow for the characterization of various CQD systems and further for their application to quantum information devices^20,21^. To date, the methodologies for the manipulation and read out of quantum states in single quantum dots have been limited to either optical methods or electrical methods; electrostatically defined quantum dots are primarily electrically controlled^6–8^, whereas bottom-up quantum dots are mainly optically controlled^22,23^. Exploring the transport properties for optically active single CQDs provides compatibility of optical and electrical manipulation of quantum states, which increases the functionality and flexibility of single-CQD devices and is expected to bring about innovation in quantum information technology.

Furthermore, CQDs form a thin film and can transport carriers when cross-linked. These properties make CQD systems excellent candidates for next-generation optoelectronic devices such as solar cells^24,25^, photodetectors^26–29^, and light-emitting devices^30,31^. Many studies on the transport properties of CQD ensembles have been reported thus far^32–41^ and have shown that ligand bridging and the morphology of the CQD array strongly influence carrier transport through CQD assembly. Characterization of the carrier transport at the single CQD level provides complementary information to that of CQD assemblies as performed by scanning tunneling microscopy/spectroscopy (STM/STS)^42,43^. It also helps in understanding the details of the carrier transport mechanism through CQDs and the role of ligands.

Among the various CQDs, lead sulfide (PbS) is one of the most attractive materials because of its broad emission/absorption spectral tunability range in the infrared regime. To date, PbS CQDs have been one of the standard materials commercially available. Additionally, they can be treated as a model material for investigation of the charge carrier transport properties in CQD assemblies, and any knowledge gained from such investigations can be translated to other CQDs of different compounds^44^. While detailed studies on PbS CQD assemblies have reported energy shell-dependent electrical conductivity properties^39,41,45,46^ and ligand bridging-dependent electron and hole mobilities^44–48^, the transport properties for single PbS CQDs have not yet been fully elucidated. Characterization of a single quantum dot can exclude the influence of size fluctuations and enables clear observation of intrinsic phenomena such as discrete energy levels and the energy shell-dependent conductivity in CQDs. For this purpose, the narrow bandgap of PbS CQDs is advantageous for the detection of a current through CQDs by nanogap metal electrodes.

In this article, we report the demonstration of single-CQD transistors based on commercially available high-quality PbS CQDs with oleic acid as a ligand. We demonstrate quantum dot size-dependent carrier transport, orbital-dependent electron charging energy and conductance, electric field modulation of the confinement potential of electrons, and spin-correlated coherent carrier transport called the Kondo effect in a PbS CQD system despite the rather long oleic acid capping. Furthermore, we find that single small-sized CQD transistors operate as SETs even at room temperature.

## Results

### Single-PbS-CQD transistors

**Fig. 1:**
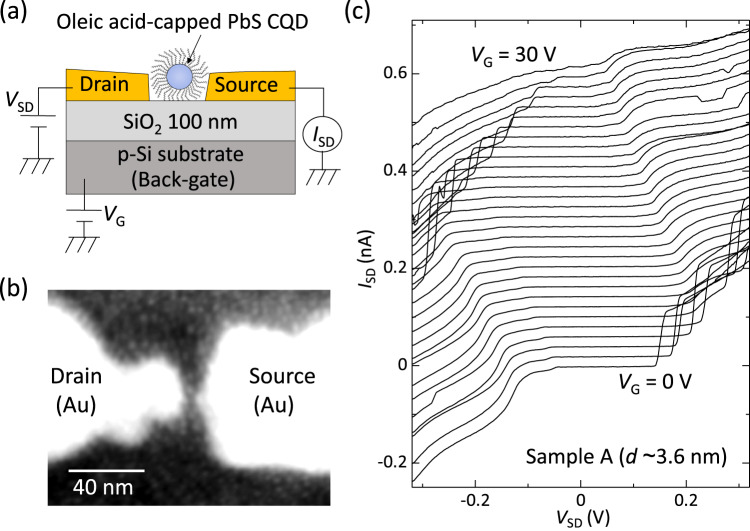
Single-PbS-CQD transistors. **a** Schematic illustration of the device structure and the experimental setup. **b** Scanning electron microscopy image of 10-nm-thick Au electrodes separated by a ~ 5 nm gap onto which PbS CQDs (diameter *d* ~ 3.6 nm) have been deposited. **c** Current–voltage (*I*_SD_–*V*_SD_) curves measured for sample A at *T* = 4 K, taken at every 1 V step of the back-gate voltage, *V*_G_, from 0 V to 30 V. The curves are offset by 0.02 nA for clarity. Only the bottom curve at *V*_G_ = 0 V corresponds to the actual scale.

**Fig. 2:**
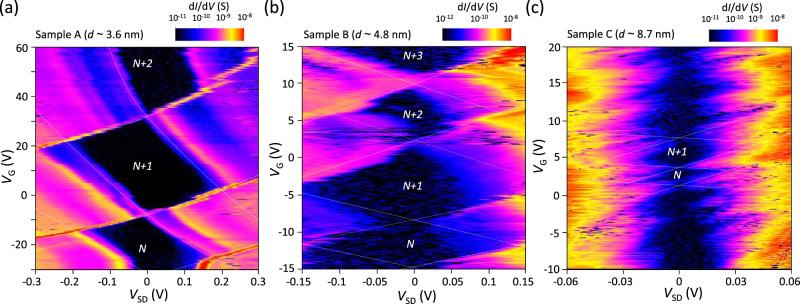
Low-temperature transport properties for samples with different CQD sizes. Coulomb stability diagrams were obtained at *T* = 4 K for samples A with diameter *d* ~ 3.6 nm (**a**), B with *d* ~ 4.8 nm (**b**), and C with *d* ~ 8.7 nm (**c**). The number of confined electrons in the CQDs is shown in each figure. The asymmetric shape of the Coulomb diamond occurs because of the asymmetric tunnel coupling between the CQD and the source-drain electrodes. The dashed lines are guides for the eyes. In (**c**), dashed lines are drawn only in the regions where the shape of Coulomb diamonds is relatively clear.

Transport measurements were performed on single PbS CQDs in contact with nanogap source-drain electrodes, as shown in Fig. 1a, b. A back-gate electrode was used to tune the electronic state of the PbS CQDs. Figure 1c shows the source-drain current, *I*_SD_, as a function of the source-drain voltage, *V*_SD_, of sample A with CQD diameter *d* ~ 3.6 nm, measured for different back-gate voltages, *V*_G_. The sample exhibits a strongly suppressed current near *V*_SD_ = 0 V, followed by a step-like current increase at high |*V*_SD_|. The voltage width of the suppressed conductance is modulated by changing *V*_G_. The suppressed current at *V*_SD_ ~ 0 V is a manifestation of the strong Coulomb interaction in this system and is called the Coulomb blockade effect. A current step occurs at a large |*V*_SD_| at which an extra electron is energetically allowed to enter the CQD. The observed *I*_SD_–*V*_SD_ characteristics clearly indicate that this sample operates as an SET. Coulomb stability diagrams were obtained by plotting the differential conductance, d*I*/d*V*_SD_, as a function of *V*_SD_ and *V*_G_, as shown in Fig. 2.

### Coulomb stability diagrams for various CQD sizes

Figure 2 shows Coulomb stability diagrams obtained for three samples with different CQD sizes (see also Supplementary Fig. S1 for more data). The dark-colored diamond-shaped areas (Coulomb diamonds) correspond to the Coulomb blockade regions. Each Coulomb diamond is associated with a well-defined number of confined electrons, *n*, in the CQD. Although the number of electrons in the CQD could not be determined, we can tell the parity of *n* from the size of the Coulomb diamonds. Especially in samples A and B, the Coulomb diamond for *n* = *N* + 1 electrons is notably larger than that for *n* = *N* and *N* + 2. The (*N* + 1) diamond contains an even number of electrons and has a larger addition energy, *E*_add_, which consists of the Coulomb charging energy, *E*_C_, and difference in orbital quantization energy, Δ*E*, in the CQD. The diamonds labeled *N* and *N* + 2 are smaller because the addition energies are only due to *E*_C_. Therefore, *N* is an odd number in these samples. In large CQDs (sample C, *d* ~ 8.7 nm), such even-odd behavior of the Coulomb diamonds is not clearly observed, as shown in Fig. 2c, most likely because of the very different numbers of electrons *n* in large CQDs; a larger PbS CQD has a narrower bandgap, as well as smaller *E*_C_ and Δ*E*, leading to electrical access to the larger *n* regime. In Fig. 2a, b and Supplementary Fig. S1a, the sizes of the Coulomb diamonds for *n* = *N* and *N* + 2 are not the same and significantly differ, indicating that *E*_C_ strongly depends on the number of electrons in these samples. Since similar even-odd behavior of the Coulomb diamonds and *n-*dependent charging energy are reported in many other few-electron quantum dot systems^9–12,49^, samples A, B, and D are in the few-electron regime, while samples C and E are in the many-electron regime.

### Charge addition energy and conductance

**Fig. 3:**
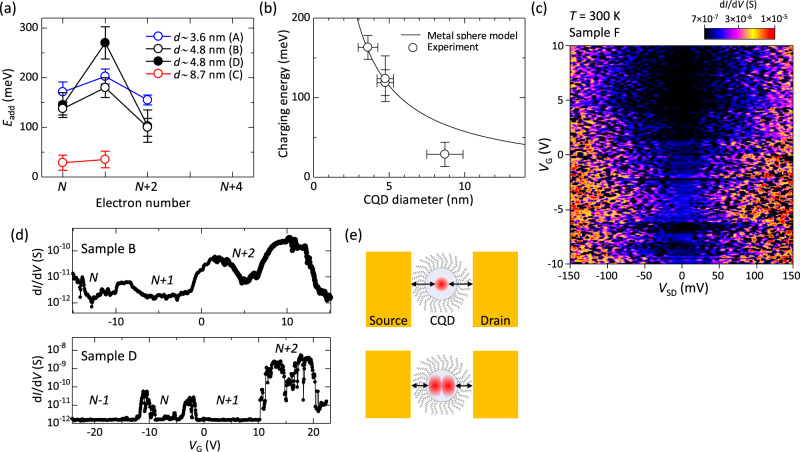
Charge addition energy and conductance. **a** Charge addition energy, *E*_add_, as a function of the number of electrons labeled in Fig. 2 and Supplementary Fig. S1. **b** Charging energy, *E*_C_, vs. CQD diameter. Open circles are experimentally obtained *E*_C_ for samples A to D, while the solid line is the calculated *E*_C_ for the metal sphere model. **c** Coulomb stability diagram obtained for sample F with *d* ~ 4.8 nm measured at room temperature. **d** Coulomb oscillation peaks for samples with *d* ~ 4.8 nm (samples B (upper) and D (lower)). The number of confined electrons in each gate voltage region is shown in each figure. **e** Schematic illustrations of electron wavefunctions in the CQDs and nanogap source-drain electrodes for s-orbitals (upper) and p-orbitals (lower). Note that the electron wavefunctions (shown as red) for higher orbitals are more extended in space, and the intrinsic gap (shown as arrows) between the metal electrodes and the electron wavefunction in the CQD changes depending on the electron orbital.

In Fig. 3a, we plot the *E*_add_ of electrons for each sample as a function of the number of electrons labeled in Fig. 2 and Supplementary Fig. S1. In small CQD samples (samples A, B, and D), *E*_add_ for *n* = *N* (odd) is slightly larger than that for *n* = *N* + 2 (odd), indicating that *E*_C_ decreases with increasing *n*. These results indicate that the electron wavefunction in the PbS CQD becomes more extended in space with increasing *n*, leading to larger capacitive coupling with the electrodes and reduced electron-electron Coulomb repulsion in the CQDs. Such an *n* dependence of *E*_add_ is not clear for large CQD samples with *d* ~ 8.7 nm; *E*_add_ shows much smaller and almost constant values in samples C and E, indicating that the spatial size of the electron wavefunction in the PbS CQDs does not significantly change and that the quantized energy levels are almost degenerate. The large peak in *E*_add_ at *n* = *N* + 1 for sample D reflects a large orbital quantization energy difference Δ*E*, which strongly depends on *n* as well as the size and shape of the measured single PbS CQDs^50^. The number of electrons in sample D might be slightly different from those in samples A and B (i.e., sample D might be probing different quantized states).

Figure 3b shows the CQD size dependence of the experimentally obtained *E*_C_. Here, *E*_C_ is plotted as the average of all *E*_add_ for *n* = odd in each sample. The calculated *E*_C_ for a metal sphere quantum dot is shown as a solid line in Fig. 3b. The self-capacitance of a metal sphere quantum dot surrounded by oleic acid is given by *C*_dot_ = 4π*ε*_0_*ε*_r_*r*, where *ε*_0_ is the vacuum permittivity, *ε*_r_ ~ 2.5 is the relative permittivity of oleic acid^51,52^, and *r* is the radius of the sphere. Then, *E*_C_ is calculated as *E*_C_ = *e*^2^/2*C*_dot_, where *e* is the elementary charge. The experimentally obtained *E*_C_ is found to be in reasonable agreement with the simple metal sphere quantum dot model. The observation of a large *E*_C_ of ~150 meV in small CQDs suggests that devices with small CQDs operate as SETs even at room temperature. Figure 3c and Supplementary Fig. S2 show Coulomb stability diagrams for three different devices (samples F, G, and H) with *d* ~ 4.8 nm measured at room temperature. The temperature dependence of the Coulomb stability diagram for sample F is also shown in Supplementary Fig. S3. While there is a tendency for the noise to increase at higher temperatures, we still obtained diamond-like Coulomb stability diagrams for samples F, G, and H, confirming that the devices operate as SETs even at room temperature. It is known that the criterion for the high-temperature operation of SETs is *E*_add_ > ~ 4*k*_B_*T*, considering thermal broadening in the single-level tunneling regime^53^, where *k*_B_ is the Boltzmann constant. Given that *E*_add_ > 120 meV for CQDs with *d* ~ 4.8 nm, which exceeds 4*k*_B_*T* at *T* = 300 K, it is reasonable to observe Coulomb diamonds even at room temperature. To date, various SET devices, such as Si-based SET structures^54–57^, carbon nanotubes^58^, graphene quantum dots^59^, and metal nanoparticles^60^, have been demonstrated to operate at room temperature. This is the demonstration of room-temperature SET operation in semiconductor nanocrystal systems. Considering that PbS CQDs are excellent emitters and absorbers of light, our device is a platform for room-temperature SETs with good optical properties, increasing the functionality and versatility of single-electron devices.

Next, we discuss the magnitude of the tunneling conductance. Figure 3d shows the differential conductance taken at *V*_SD_ = 0 V as a function of *V*_G_ plotted for samples with *d* = 4.8 nm (i.e., samples B and D), exhibiting clear Coulomb oscillation peaks. The conductance at the Coulomb peaks shows a strong dependence on *n*. The two paired *N-*th and (*N* + 1)th Coulomb peaks exhibit almost the same conductance and one order of magnitude lower conductance than the other paired (*N* + 2)-th and (*N* + 3)-th peaks located in the high *V*_G_ region (this tendency is clearer in sample D). These results strongly suggest that the *N* and (*N* + 1) electrons ((*N* + 2) and (*N* + 3) electrons) in these CQDs are in spin-up and spin-down states while occupying the same orbital. As mentioned in the previous paragraph, the spatial size of the electron wavefunction becomes larger for higher orbitals. These facts lead to the conclusion that the tunneling barrier in the present metal electrode-CQD system is formed not only by the capping materials (oleic acid) but also by the intrinsic gaps between the metal electrodes and the electron wavefunction in the CQD, as schematically illustrated in Fig. 3e. Since the tunneling conductance exponentially depends on the tunneling gap, even a small change in the size of the electron wavefunction gives rise to a large difference in the tunneling conductance.

### Kondo effect in PbS CQDs

**Fig. 4:**
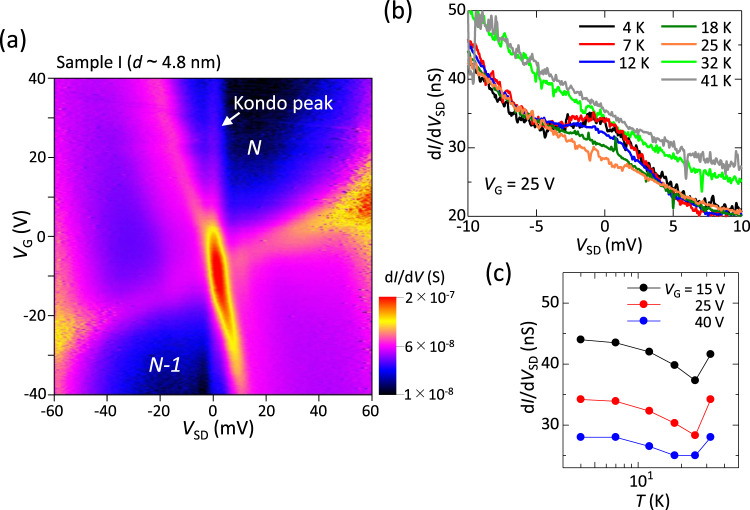
Kondo effect in single PbS CQDs. **a** Coulomb stability diagram obtained for high-conductance sample I with *d* ~ 4.8 nm. **b** Differential conductance as a function of *V*_SD_ measured at *V*_G_ = 25 V for various temperatures. **c** Temperature dependence of d*I*/d*V*_SD_ at *V*_SD_ = 0 V for various *V*_G_.

Next, let us examine the spin-dependent carrier transport observed in single PbS CQDs. Figure 4a shows a Coulomb stability diagram for a high-conductance sample with *d* ~ 4.8 nm (sample I) measured at 4 K. One can only observe the data near the charge degeneracy point of the Coulomb stability diagram for the addition of the *N*-th electron owing to the poor gate modulation efficiency in this sample. The Coulomb diamond for *n* = *N* shows a clear resonant enhancement of the zero-bias conductance. This behavior arises from the formation of a spin singlet state between an unpaired electron in the CQD and an electron with the opposite spin in the electrodes, i.e., the spin-half Kondo effect^61,62^. The spin-half Kondo effect only appears in *n* = odd Coulomb diamonds, suggesting that *N* is an odd number in Fig. 4a.

In Fig. 4b, d*I*/d*V*_SD_ is plotted as a function of *V*_SD_ at *V*_G_ = 25 V for various temperatures. The Kondo peak at *V*_SD_ = 0 V grows with decreasing temperature. Figure 4c shows the temperature dependence of d*I*/d*V*_SD_ at *V*_SD_ = 0 V (i.e., at the Kondo peak) for various *V*_G_ values. With increasing temperature, d*I*/d*V*_SD_ at *V*_SD_ = 0 V decreases up to ~30 K because of the weakened Kondo effect and then begins to increase at *T* > 30 K due to the increased background conductance induced by the thermal broadening of the adjacent tunneling peak. Although the background conductance due to the falling edges of the adjacent tunneling peaks hinders precise determination of the full-width at half-maximum of the Kondo peak, *w*, it is roughly estimated to be *ew* ~ 3 meV. The Kondo temperature, *T*_K_, is expressed as *k*_B_*T*_K_ ~ *ew*^61,63,64^. From this relation, *T*_K_ in this sample is determined to be *T*_K_ ~ 30 K, which is consistent with the enhanced conductance at *V*_SD_ = 0 V for *T* < 30 K in Fig. 4c.

## Discussion

In sample A, the slopes of the Coulomb diamond boundaries are not constant and depend on *V*_G_ and *V*_SD_ (see Fig. 2a and Supplementary Fig. S4a), indicating that the electron density profile in the CQD is affected by the potential gradient induced by *V*_G_ and *V*_SD_. In this sample, the noise level is low enough to identify the excited-state lines in the Coulomb stability diagram^65^. Clear excited-state lines are observed, from which the orbital quantization energy differences for *n* electrons, Δ*E*(*n*), are determined to be Δ*E*(*N*) = 50 meV, Δ*E*(*N* + 1)_low_ = 80 meV from the low *V*_G_ region of the (*N* + 1) Coulomb diamond, Δ*E*(*N* + 1)_high_ = 90 meV from the high *V*_G_ region of the (*N* + 1) Coulomb diamond, and Δ*E*(*N* + 2) = 35 meV (see Supplementary Fig. S4). We simulated Δ*E* for cubic PbS quantum dots with similar volumes in the few-electron regime using the software QTCAD from Nanoacademic Technologies^66^ and found a Δ*E* of 10-100 meV, which is in reasonable agreement with the experimentally observed Δ*E*(*n*). Although the observed Δ*E*(*N* + 1)_high_ and Δ*E*(*N* + 1)_low_ originate from the same excited state, Δ*E*(*N* + 1)_high_ is larger than Δ*E*(*N* + 1)_low_, indicating stronger confinement of electrons at higher *V*_G_. Furthermore, Δ*E*(*N*) significantly increases with increasing *V*_G_ (see Supplementary Fig. S4 and Note 1), which again indicates stronger confinement of electrons in the CQD at higher *V*_G_. These results indicate the strong influence of the external electric field induced by *V*_G_ and *V*_SD_ on the confinement potential of electrons in single PbS CQDs, suggesting the tunability of the effective confinement size of electrons and the bandgap in CQDs by the external electric field.

Regarding the Kondo effect, we found a sample (sample J, *d* ~ 4.8 nm) that has higher conductance than sample I, as shown in Supplementary Fig. S5. The width of the Kondo peak in sample J is *ew* ~ 7 meV at *V*_G_ = 40 V, suggesting a very high Kondo temperature of *T*_K_ ~ 80 K. The *T*_K_ in a SET is described by ${k}_{{{{{{\rm{B}}}}}}}{T}_{{{{{{\rm{K}}}}}}} \sim {({E}_{{{{{{\rm{C}}}}}}}{\it\Gamma })}^{\frac{1}{2}}$ exp[-π(*μ*-*ε*_0_)/2*Γ*], where *Γ* is the tunnel coupling energy between the quantum dot and the source-drain electrodes, *μ* is the electrochemical potential of the source-drain electrodes at *V*_SD_ = 0 V, and *ε*_0_ is a spin-degenerate energy level in the quantum dot occupied by a single electron^61^. Therefore, *T*_K_ is limited by both *E*_C_ and *Γ*. Considering the large charging energy *E*_C_ ~ 120 meV (corresponding to a thermal energy of 1400 K) in PbS CQDs with *d* ~ 4.8 nm, the small *Γ* limits the *T*_K_ in samples I and J; this is reasonable because oleic acid (~2 nm long ligand) is known as a long-chain insulating ligand that blocks charge carrier transport through CQDs^44^. The observation of a high *T*_K_ in oleic acid-capped PbS CQDs suggests the feasibility of realizing a much higher *T*_K_ up to room temperature by replacing oleic acid with short-chain ligands, which may add the Kondo effect as one of the carrier transport mechanisms in CQD assemblies operated at room temperature.

In summary, we have demonstrated single-CQD transistors based on commercially available high-quality PbS CQDs with oleic acid as a ligand. The transport characteristics strongly depend on the quantum dot size; a few-electron regime is observed in small PbS CQDs, while a many-electron regime is observed in large CQDs. From the *n* dependence of *E*_C_ and the conductance, we demonstrated that the tunneling barrier in this system is formed not only by the capping material but also by the intrinsic gap between the electron wavefunction in the CQDs and electrodes. Analysis of the excited states indicates that the confinement potential of electrons in CQDs is strongly affected by the external electric field induced by *V*_G_ and *V*_SD_. The Kondo effect is also observed in a single-CQD system; this indicates strong coupling between the electrodes and the CQDs despite the use of a long-chain insulating oleic acid ligand. These results provide nanoscopic insight into carrier transport through CQDs at the single quantum dot level, which is essential for developing CQD applications in optoelectronic devices, such as solar cells and photodetectors. Furthermore, the large charging energy in small CQDs enables SET operation even at room temperature. Considering that PbS CQDs are excellent emitters and absorbers of light, our device is a platform for room-temperature SETs with good optical properties, increasing the functionality and versatility of single-electron devices. This will bring about innovation in quantum information technology.

## Methods

### Sample preparation

We first formed thin gold nanojunctions on a 100-nm-thick SiO_2_ layer grown on a *p*-type Si substrate. The thickness and width of the gold nanojunctions were ~ 10 nm and ~60 nm, respectively. The *p*-type Si substrate was used as a back-gate electrode. Then, nanogap source-drain electrodes with gap sizes of 3-10 nm were fabricated using the electrical break junction (EBJ) method^67,68^ (see Supplementary Fig. S6). The gap sizes of the fabricated nanogap electrodes were characterized by scanning electron microscopy (SEM): approximately 10% of them have a nanogap size of <5 nm, and 50% of them have a gap size of 5-10 nm, as shown in Supplementary Fig. S6c. These gap sizes are suitable for detecting a current through single PbS CQDs in this study. The formation of thin gold nanojunctions, wire bonding, and fabrication of nanogap source-drain electrodes using the EBJ method were all performed in ambient air. After nanogap formation, we placed the devices with nanogap electrodes into a glovebox or a glove bag filled with nitrogen gas and drop-casted a solution of PbS CQDs (10 mg/mL) onto the nanogap electrodes, followed by blowing off of the excess solution using dry nitrogen gas. We then mounted the samples in a cryostat insert under a nitrogen gas atmosphere. We introduced them into a top-loading 4 K cryostat while maintaining a nitrogen gas environment. After introducing the samples into the cryostat, we replaced the nitrogen gas with helium gas and conducted measurements in a helium gas environment. During these procedures, we paid particular attention to not exposing PbS CQDs to air to avoid physisorption of water/moisture and oxygen^69^. The fabrication yield of single PbS transistors is very low and typically a few %. An SEM image of a typical PbS CQD sample is shown in Fig. 1b and Supplementary Fig. S7. One can observe PbS CQDs around and between the nanogap source-drain electrodes. PbS CQDs are also distributed on the Au electrodes but cannot be observed due to the off-focus and low contrast.

Transport measurements were performed mostly at cryogenic temperatures on three types of commercial PbS CQDs (Supplementary Fig. S8): oleic acid-capped PbS CQDs with 1100 nm peak emission (*d* ~ 3.6 ± 0.7 nm), oleic acid-capped PbS CQDs with 1500 nm peak emission (*d* ~ 4.8 ± 0.6 nm), both in toluene solution from Sigma‒Aldrich Co. LLC, and oleic acid-capped PbS CQDs with 2000 nm peak absorption (*d* ~ 8.7 ± 1.2 nm), in octane solution from Quantum Solutions. In this paper, we introduce eleven PbS CQD samples: a PbS CQD with *d* ~ 3.6 nm (sample A), eight CQDs with *d* ~ 4.8 nm (samples B, D, F, G, H, I, J, and K), and two CQDs with *d* ~ 8.7 nm (samples C and E).

### Determination of the charge addition energies and the charging energies

From Fig. 2 and Supplementary Fig. S1, we derived *E*_add_ for each *n* through the following procedures. The difference in the electrochemical potential between the source and drain electrodes where the tunneling-in and tunneling-out lines of a Coulomb diamond cross (i.e., *e* | *V*_SD_ (apex)| at the apex of each Coulomb diamond in Supplementary Fig. S9) is equal to the addition energy. When |*V*_SD_ (apex)| was polarity dependent, the size of the Coulomb diamonds was determined as the average of the two values. *E*_C_ was determined from the addition energy for *n* = odd. In Fig. 3b, *E*_C_ was determined by the average of all *E*_add_ for *n* = odd.

For most of the samples, the Coulomb stability diagrams appear noisy, probably because of a sudden change in the electrostatic environment of the measured CQDs induced by the charge fluctuations in neighboring CQDs. We observed larger noise in larger CQDs, probably because of the frequent trapping/detrapping of electrons in the surrounding CQDs due to the smaller *E*_C_ and Δ*E* in larger CQDs. Therefore, we could not discuss *E*_add_ in detail for large CQD samples (i.e., samples C and E). We observed clear charge degeneracy points in the Coulomb stability diagrams, which contradicts the previously reported results for large PbS CQDs^3^; the reason for the contradiction is unknown but seemingly caused by electron transport through serially coupled double CQDs in ref. ^3^.

In our samples, the CQD, acting as a Coulomb island, is surrounded by other CQDs and electrodes as shown in Fig. 1b and Supplementary Fig. S7. In this configuration, the actual capacitance should always be larger than the self-capacitance of a metal sphere quantum dot because the Coulomb island is capacitively coupled with the electrodes and the surrounding CQDs, leading to screening of the electric field^70^. This could result in a charging energy smaller than that of the metal sphere model. However, in the few-electron regime of small CQD samples, the spatial extension of electron wavefunctions is smaller than the geometrical CQD size estimated from the SEM images, as illustrated in Fig. 3e. Therefore, the agreement between the experimentally obtained *E*_C_ in small CQDs and the metal sphere model could be the result of the compensation of both the screening effect of the surroundings and the reduced effective CQD size in the few-electron regime. In connection with this discussion, we elaborate on the contribution of ligands. In this study, the CQDs are capped with long-chain insulating oleic acid (length ∼ 2 nm) as an organic ligand, which results in weak interactions (weak capacitive coupling) with neighboring electrodes/CQDs and minimal wavefunction overlap with the neighboring electrodes/CQDs, leading to more isolated CQDs that can be explained by the almost isolated metal sphere model.

### Analysis of Coulomb stability diagrams for samples C and E (*d* ~ 8.7 nm)

The Coulomb diamond signatures appear particularly unclear in Fig. 2c and Supplementary Fig. S1b (i.e., samples C and E). While some Coulomb diamonds do not close, suggesting the possibility of multi-dot behavior, the possibility that in this system, the significant noise and instability of the characteristics may be influenced by the charge fluctuations in the surrounding CQDs should also be considered. By observing the structure of the devices for samples C and E using SEM, we found that the gap size of the nanogap electrode in both samples was approximately 10 nm. Considering the dot size of *d* ∼8.7 nm (~12 nm including oleic acid ligand), parallel quantum dot conduction is more likely to be detected than serial quantum dot conduction in this situation. In the case of parallel quantum dot arrangements, we would observe overlap of two types of Coulomb diamonds. However, such behavior is not clearly observed in Fig. 2c and Supplementary Fig. S1b. Therefore, we think that the unclear Coulomb diamonds are likely due to the significant charge fluctuations in the surrounding CQDs.

As mentioned in the main text, we believe the results for samples C and E correspond to measurements of the many-electron regime. In general, single-electron transport in the many-electron regime is well described by the constant interaction model^9^. Therefore, in the analysis of Coulomb diamonds in Fig. 2c, we arranged the lines forming the boundaries of the diamonds (two types of lines with positive and negative slopes) such that they exhibit a gate-independent constant slope, allowing us to determine the size of the diamonds.

### Observed carrier type

In samples B and D, *E*_C_ decreases while the conductance increases with increasing *V*_G_ (see Fig. 2b and Supplementary Fig. S1a), which is typical for SETs in the few-electron transport regime^9–12,49^. We also observed a bandgap region near the conduction band edge in sample K (see Supplementary Fig. S10). These results suggest that we are measuring electron transport in the conduction band and that electrons are being added to the conduction band of the PbS CQDs with increasing *V*_G_. Only in sample A does the conductance slightly decrease, while *E*_C_ decreases with increasing *V*_G_. The reason for this behavior of the conductance in sample A is unknown thus far.

### Supplementary information

Supplementary Information

Peer Review File

## Data Availability

The measurement and analyses data generated in this study have been deposited in the Figshare database under accession code 10.6084/m9.figshare.24311608. They are also available from the corresponding author upon reasonable request.
