# Supplementary material for: Single PbS colloidal quantum dot transistors
Source: Nat Commun. 2023 Nov 18;14:7486. doi: 10.1038/s41467-023-43343-7 (PMC10657373; doi:10.1038/s41467-023-43343-7)
Supplement: Supplementary file 1 — Supplementary Information [file 41467_2023_43343_MOESM1_ESM.pdf]

Supplementary information

Single PbS colloidal quantum dot transistors

Kenji Shibata,[1,†] Masaki Yoshida,[1] Kazuhiko Hirakawa,[2,3] Tomohiro Otsuka,[4,5,6,7,8] Satria Zulkarnaen Bisri,[9,10] and Yoshihiro Iwasa[9,11]

[1]*Department of Electrical and Electronic Engineering, Tohoku Institute of Technology, 35-1 Yagiyama, Kasumi-cho, Taihaku-ku, Sendai, 982-8577, Japan*

[2]*Institute of Industrial Science, University of Tokyo, 4-6-1 Komaba, Meguro-ku, Tokyo 153-8505, Japan*

[3]*Institute for Nano Quantum Information Electronics, University of Tokyo, 4-6-1 Komaba, Meguro-ku, Tokyo 153-8505, Japan*

[4]*Research Institute of Electrical Communication, Tohoku University, 2-1-1 Katahira, Aoba-ku, Sendai, 980-8577, Japan*

[5]*WPI Advanced Institute for Materials Research, Tohoku University, Sendai 980-8577, Japan*

[6]*Department of Electronic Engineering, Tohoku University, Aoba 6-6-05, Aramaki, Aoba-Ku, Sendai 980-8579, Japan*

[7]*Center for Science and Innovation in Spintronics, Tohoku University, 2-1-1 Katahira, Aoba-ku, Sendai 980-8577, Japan*

[8]*Quantum Functional System Research Group, RIKEN Center for Emergent Matter Science, 2-1 Hirosawa, Wako, Saitama 351-0198, Japan*

[9]*Emergent Device Research Team, RIKEN Center for Emergent Matter Science, 2-1 Hirosawa Wako, Saitama 351-0198, Japan*

[10]*Department of Applied Physics and Chemical Engineering, Tokyo University of Agriculture and Technology, 2-24-16 Naka-cho, Koganei-shi, Tokyo 184-8588, Japan*

[11]*Department of Applied Physics and Quantum-Phase Electronics Center, University of Tokyo, Hongo, Bunkyo-ku, Tokyo, 113-8656, Japan*

---

†Corresponding Author's Email: kshibata@tohtech.ac.jp

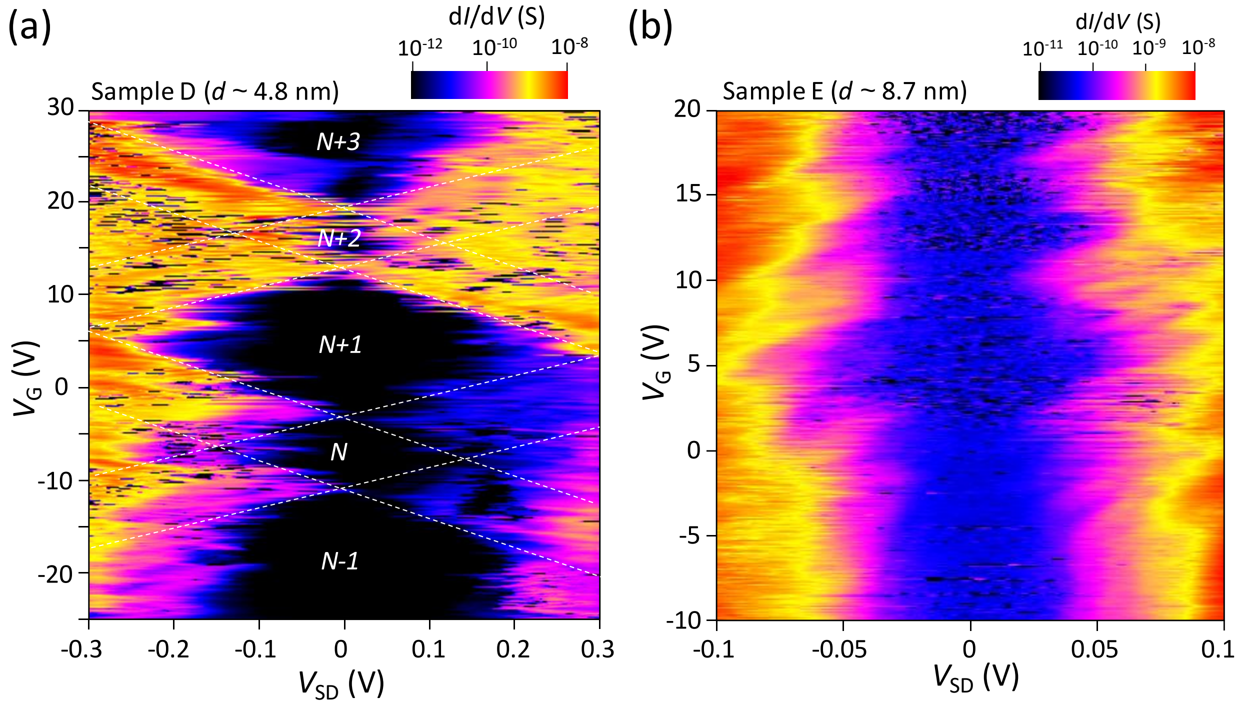

Supplementary Figure S1: Low-temperature transport properties for samples D and E.

Coulomb stability diagrams obtained for samples (a) D with $d \sim 4.8$ nm and (b) E with $d \sim 8.7$ nm. The number of confined electrons in the CQD is shown in each figure. The dashed lines are guides for the eyes. While dashed lines are not drawn for sample E due to the ambiguous Coulomb diamonds, the size of Coulomb diamonds in sample E would be comparable to that in sample C.

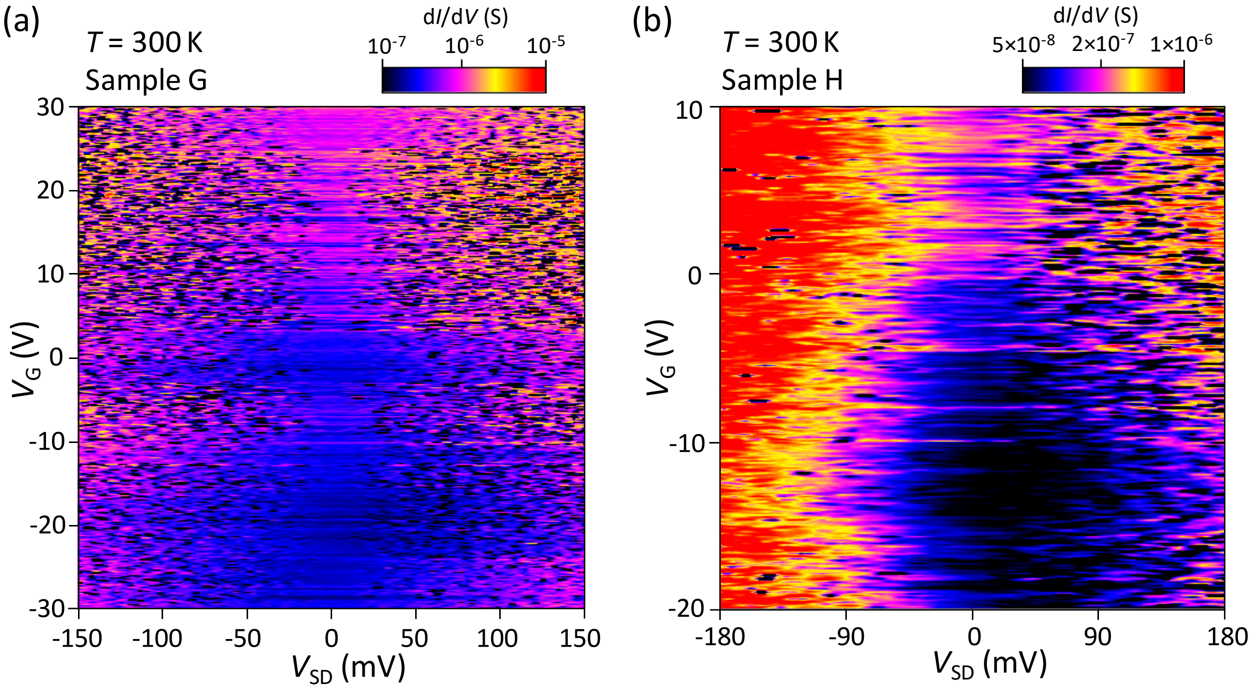

Supplementary Figure S2: Room-temperature transport properties for samples with *d* ~ 4.8 nm.

Coulomb stability diagrams for samples (a) G and (b) H with d ~ 4.8 nm measured at room temperature T = 300 K. While there is a tendency for the noise to increase at higher temperatures, we still obtained diamond-like Coulomb stability diagrams, confirming that the devices operate as SETs even at room temperature.

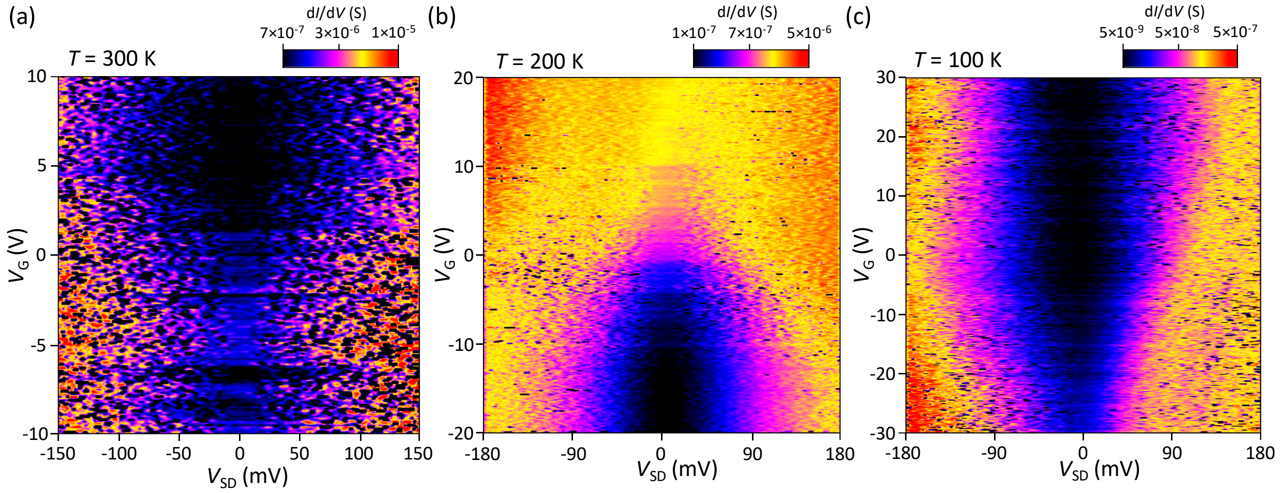

Supplementary Figure S3: Temperature dependence of the transport properties for sample F (d ~ 4.8 nm). Coulomb stability diagrams for sample F with d ~ 4.8 nm measured at high temperatures of (a) T = 300 K, (b) 200 K, and (c) 100 K. The transport characteristics significantly vary at different temperatures, probably due to the influence of thermal shrinkage/expansion and/or changes in the amount of surface charge. However, we observed part of diamond-like Coulomb stability diagrams at all temperatures, suggesting that the sample operates as a SET up to room temperature. With increasing temperature, the conductance of the sample increases due to thermally assisted tunneling. The sample becomes insulating in the low V_{SD} region at T = 4 K.

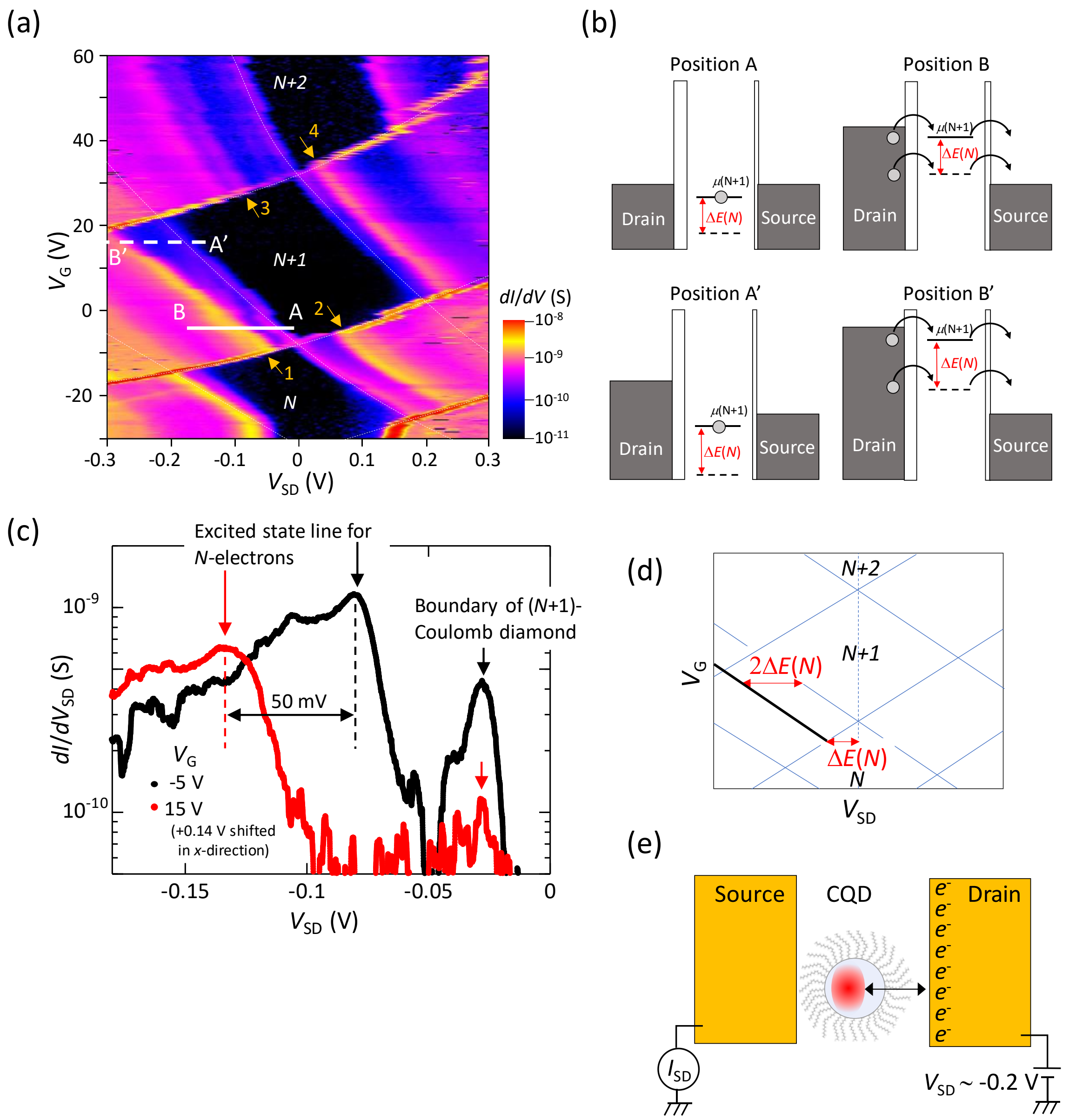

Supplementary Figure S4: Detailed analysis of the excited states in sample A.

(a) Coulomb stability diagram obtained for sample A. The positions of the excited-state lines are indicated by orange arrows with assigned numbers 1, 2, 3, and 4, which correspond to $\Delta E(N)$, $\Delta E(N+1)_{low}$, $\Delta E(N+1)_{high}$, and $\Delta E(N+2)$ in the main text, respectively. (b) Schematic illustrations of the band diagrams at positions A (upper left), B (upper right), A' (lower left), and B' (lower right) in (a), where $\mu(N+1)$ is the electrochemical potential for adding the (N+1)-th electron. $\Delta E(N)$ is the ground- to excited-state energy separation for N electrons. The dashed line indicates the electrochemical potential due to the excited state of N electrons. (c) Differential conductance as a function of V_{SD} along the solid line AB (black curve, V_G = -5 V) and the dashed line A'B' (red curve, V_G = 15 V). Data for V_G

= 15 V are shifted by 0.14 V in the *x*-direction so that the conductance peaks determined by the boundary of the (N+1) Coulomb diamond match for comparison of the voltage spacing between the boundary of the (N+1) Coulomb diamond and the excited-state line of the N electrons. (d) Schematic illustration of the orbital quantization energy differences for N electrons, $\Delta E(N)$, derived from the excited-state line. (e) Schematic illustrations of electron wavefunctions (shown as red) in the CQDs and nanogap source-drain electrodes under a high source-drain voltage of V_{SD} ~ 0.2 V. See also Supplementary Note 1 for detailed explanations.

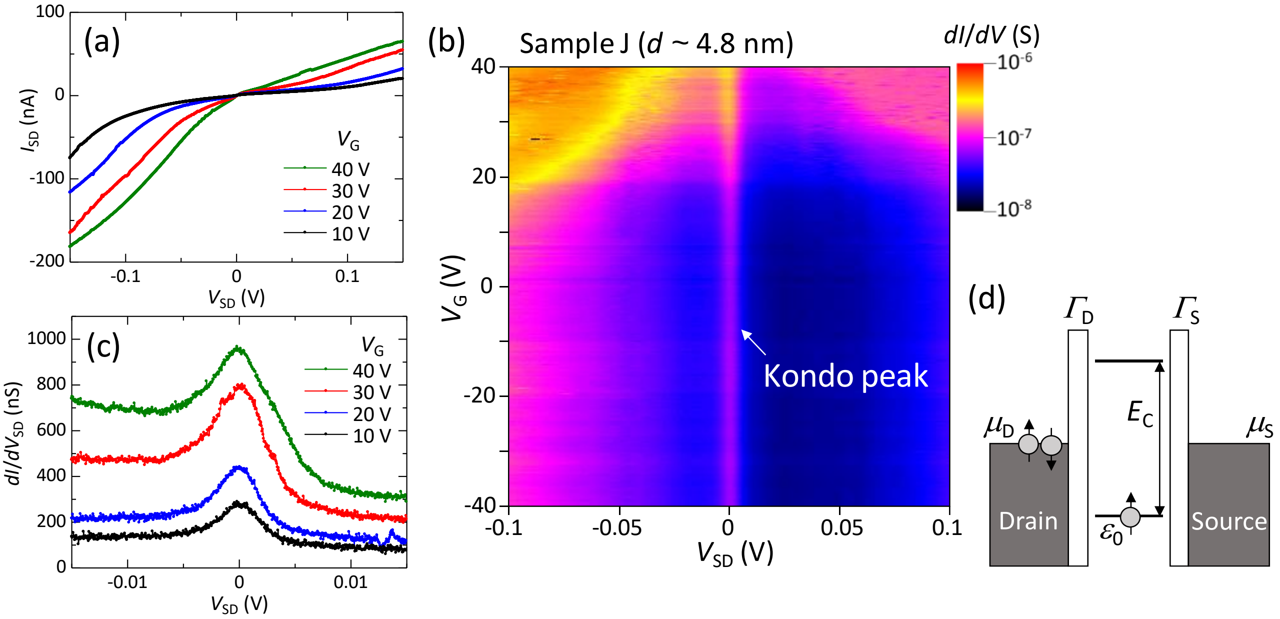

Supplementary Figure S5: Kondo effect in sample J (d ~ 4.8 nm) measured at T = 4 K.

(a) I-V curves plotted for various V_G values. The conductance is almost one order of magnitude higher than that in sample I. (b) Coulomb stability diagram of sample J. A clear Kondo peak is observed at V_{SD} = 0 V. (c) Differential conductance as a function of V_{SD} (i.e., Kondo peak) plotted for various V_G values. The width of the Kondo peak, w, depends on V_G; w becomes larger when V_G approaches the charge degeneracy point. Such behavior is known to be typical of the Kondo effect in quantum dot systems. From the width of the Kondo peak, the Kondo temperature T_K is estimated to be T_K ~ 50 K at V_G = 10 V and T_K ~ 80 K at V_G = 40 V. (d) Schematic band diagram of a quantum dot with one spin-degenerate energy level ε_0 occupied by a single electron, where Γ_D and Γ_S are the tunnel coupling energies to the drain and source electrodes, respectively. The energy states of the source and drain electrodes are filled up to electrochemical potentials μ_D and μ_S, respectively. The tunnel coupling energy, Γ, between the quantum dot and the source-drain electrodes is calculated by $\Gamma = \Gamma_D + \Gamma_S$, while the electrochemical potential of the source-drain electrodes at V_{SD} = 0 V is expressed as $\mu_D = \mu_S = \mu$ in the main text.

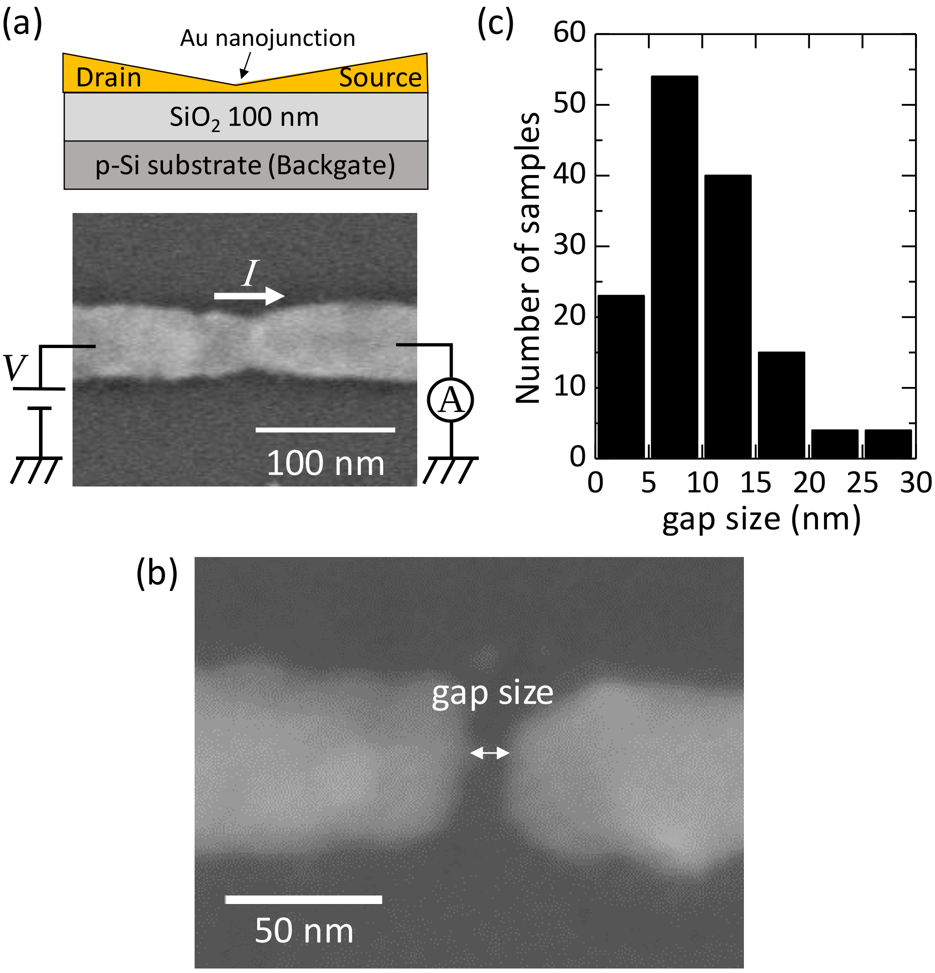

Supplementary Figure S6: Fabrication of nanogap source-drain electrodes by the electrical break junction (EBJ) method.

(a) Schematic cross-sectional image (upper) and SEM image (top view) of a sample along with the experimental setup for the EBJ method (lower). (b) Typical nanogap source-drain electrode formed by the EBJ method. (c) Histogram of the fabricated nanogap sizes.

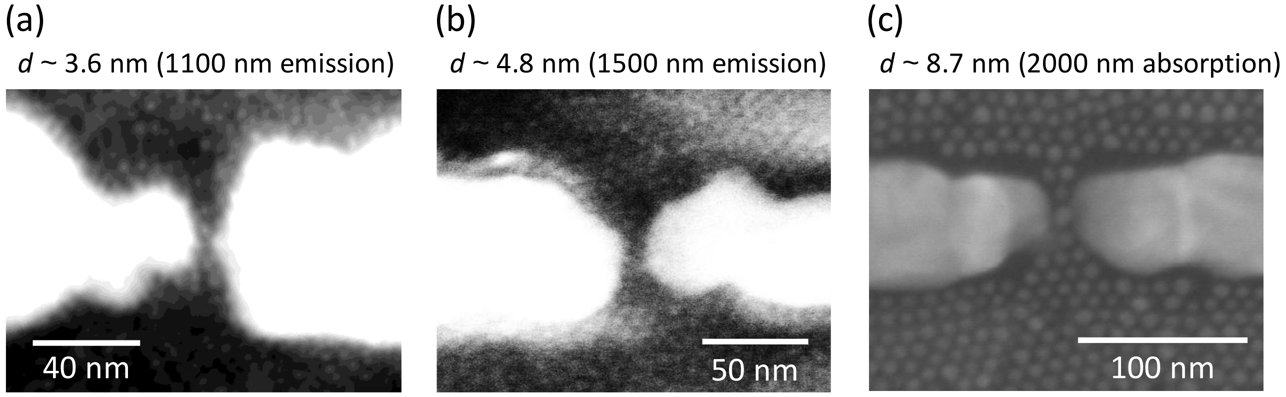

Supplementary Figure S7: SEM images of fabricated single-PbS-CQD transistors

SEM images of typical single-PbS-CQD transistors (top view) for diameter d values of (a) $d = 3.6$ nm, (b) $d = 4.8$ nm, and (c) $d = 8.7$ nm.

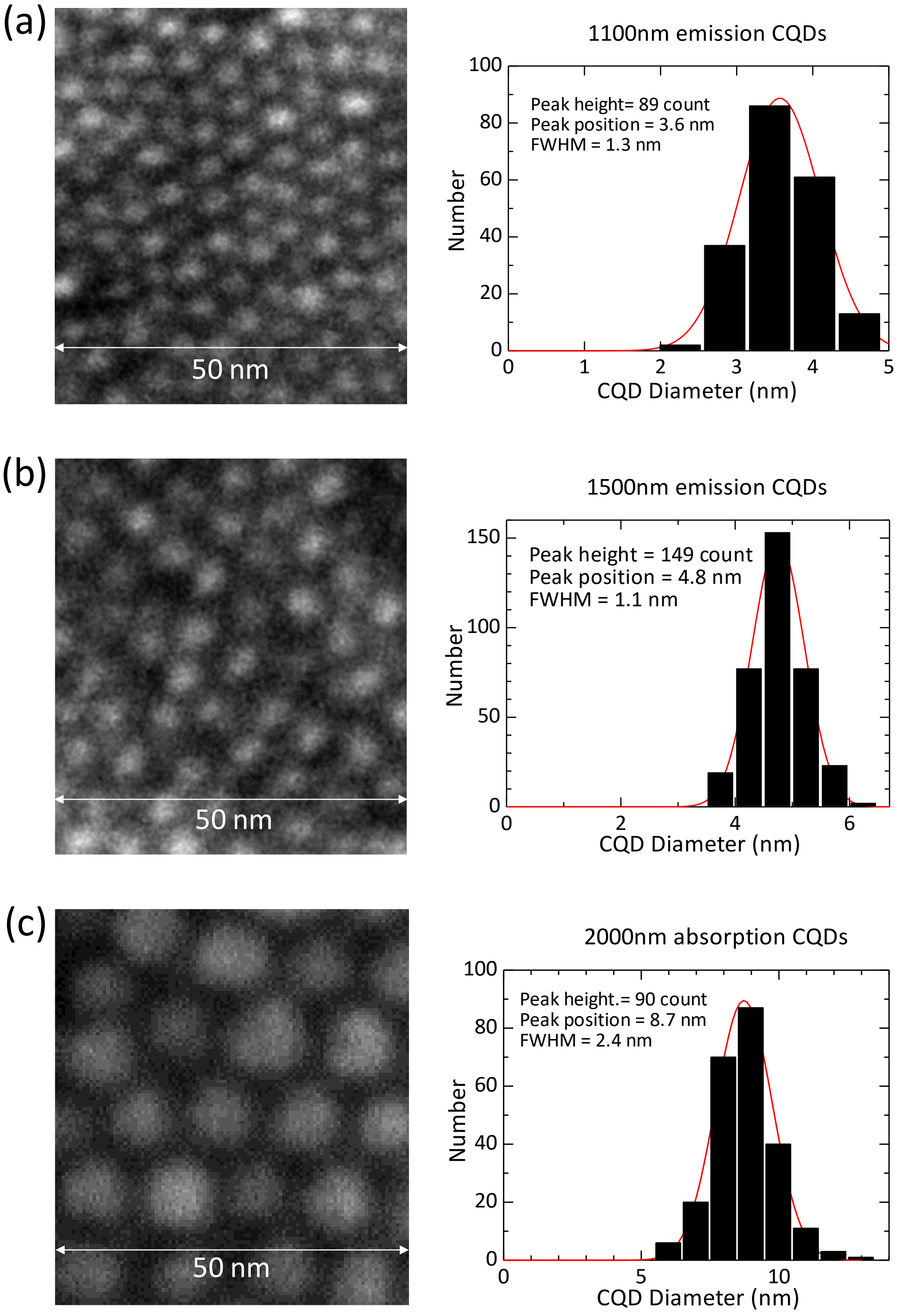

Supplementary Figure S8: Size distribution of PbS CQDs.

SEM image (left) and histogram of the diameters with the Gaussian fitting result shown as a red line (right) for oleic acid-capped PbS CQDs with (a) 1100 nm peak emission ($d \sim 3.6$ nm), (b) 1500 nm peak emission ($d \sim 4.8$ nm), and (c) 2000 nm peak absorption ($d \sim 8.7$ nm).

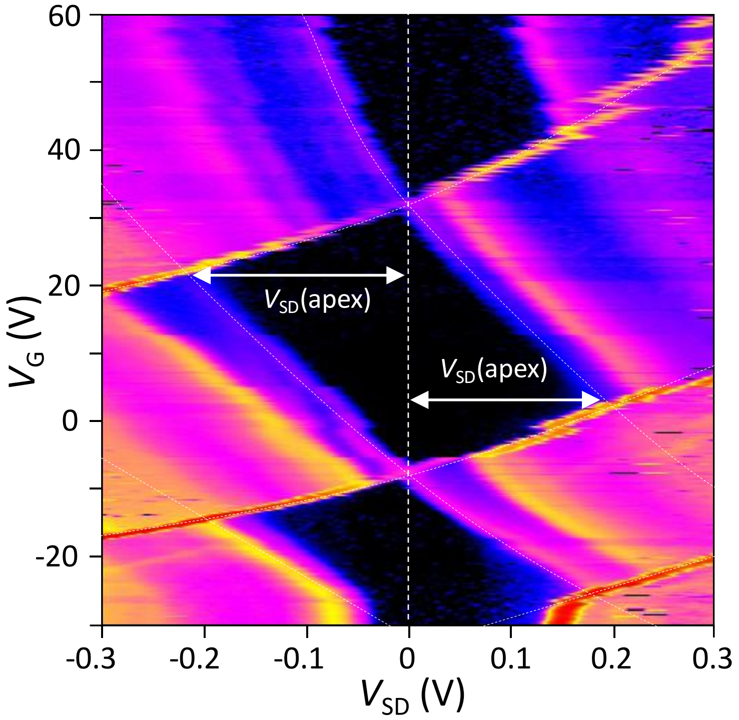

Supplementary Figure S9: Determination of the addition energy, E_{add}, for each Coulomb diamond.

The E_{add} for each Coulomb diamond is determined by $e|V_{SD}(apex)|$, where e is the elementary charge. When $|V_{SD}(apex)|$ is polarity dependent, E_{add} is determined as the average of the two values.

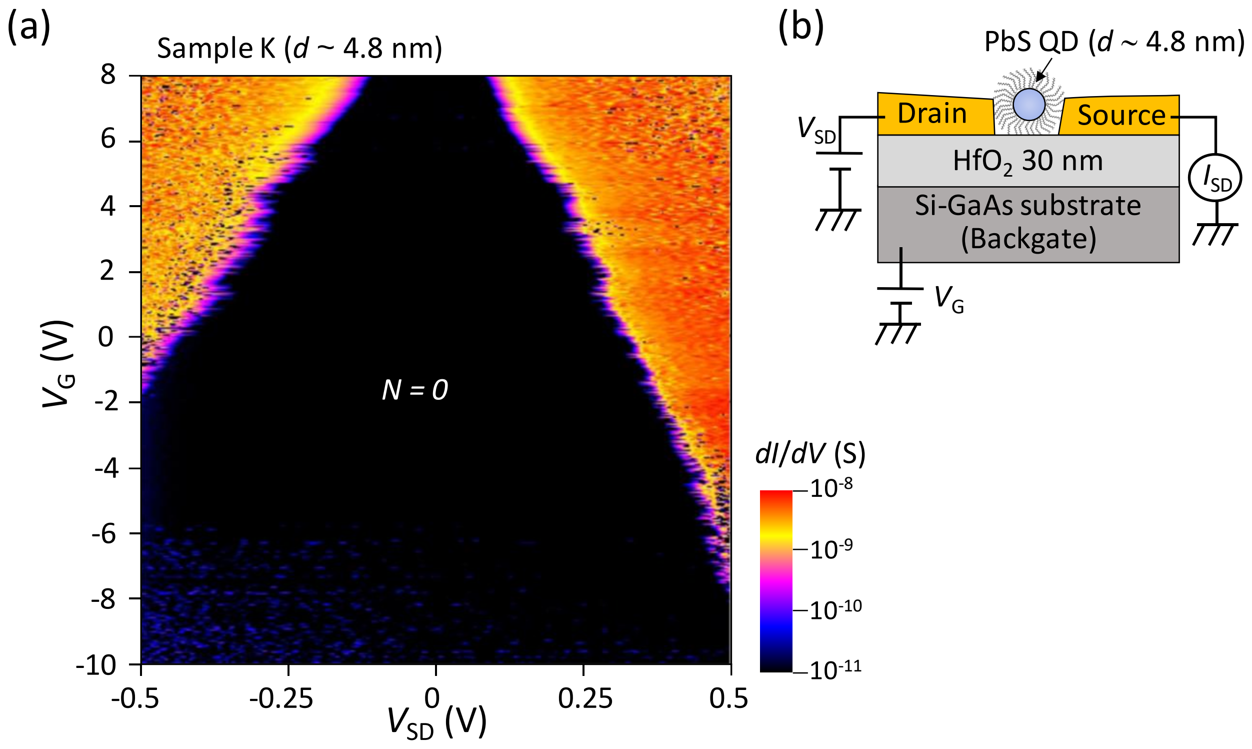

Supplementary Figure S10: Observation of the bandgap region.

(a) Coulomb stability diagram for PbS CQD sample K (*d* ~ 4.8 nm) with HfO_2 as the gate dielectric. We observed that the large Coulomb gap monotonically increases with increasing negative V_G, suggesting the bandgap region of the PbS CQD. Since the Coulomb gap decreases with increasing positive V_G, we detect the bandgap region near the conduction band edge. The reason for the bandgap observation in this sample is the slight difference in the amount of surface charge caused by the use of a different gate dielectric. (b) Schematic cross-sectional image of the PbS CQD sample with HfO_2 as the gate dielectric.

Supplementary Note 1

Analysis of the excited states in sample A.

Supplementary Fig. S4a shows the Coulomb stability diagram obtained for sample A. The positions of the excited-state lines are indicated by orange arrows with assigned numbers 1, 2, 3, and 4, which correspond to $\Delta E(N)$, $\Delta E(N+1)_{low}$, $\Delta E(N+1)_{high}$, and $\Delta E(N+2)$ in the main text, respectively. Supplementary Fig. S4b shows schematic illustrations of the band diagrams at positions A (upper left), B (upper right), A' (lower left), and B' (lower right) in (a), where $\mu(N+1)$ is the electrochemical potential for adding the (N+1)-th electron. $\Delta E(N)$ is the ground- to excited-state energy separation for N electrons. The dashed line indicates the electrochemical potential due to the excited state of N electrons. At positions B and B', electrochemical potentials in the CQD are pushed up by the electric field from V_{SD} and enter the bias window with an increase in the negative V_{SD}, resulting in electrons being able to be transported through either the ground state of (N+1) electrons (solid line) or the excited state of N electrons (dashed line). Supplementary Fig. S4c shows the differential conductance as a function of V_{SD} along the solid line AB (black curve, V_G = -5 V) and the dashed line A'B' (red curve, V_G = 15 V). Data for V_G = 15 V are shifted by 0.14 V in the x-direction so that the conductance peaks determined by the boundary of the (N+1) Coulomb diamond match for comparison of the voltage spacing between the boundary of the (N+1) Coulomb diamond and the excited-state line of the N electrons. The voltage difference between the boundary of the (N+1) Coulomb diamond and the excited-state line of the N electrons shows a significant difference between the two datasets, suggesting that $\Delta E(N)$ increases with increasing V_G. Such behavior is not observed in the positive V_{SD} region, probably because of the asymmetric tunnel coupling in this sample; the tunnel coupling to the source is strong, whereas that to the drain is weak, leading to the polarity-dependent appearance of the anomaly. Supplementary Fig. S4d shows schematic illustration of the orbital quantization energy differences for N electrons, $\Delta E(N)$, derived from the excited-state line. The voltage difference between the boundary of the (N+1) Coulomb diamond and the excited-state line of the N electrons corresponds to ~ $2\Delta E(N)$, indicating that $\Delta E(N)$ increases by ~ 25 meV with the increase in V_G from V_G = -5 V to 15 V. Supplementary Fig. S4e shows schematic illustrations of electron wavefunctions (shown as red) in the CQDs and nanogap source-drain electrodes under a high source-drain voltage of V_{SD} ~ 0.2 V. The main reason for the significant change in $\Delta E(N)$ (i.e., confinement size) for sample A is likely the large negative V_{SD} applied at the

dashed line A'B'. The energy scale is very large in sample A due to the small quantum dot size, and at the dashed line A'B', a negative voltage of 0.1-0.3 V is applied. In this case, a strong electric field from the nanogap metal electrodes (with an electric field intensity of approximately 0.2 MV/cm for an applied voltage of 0.2 V and a nanogap electrode gap size of 10 nm) is applied to the CQD, leading to modulation of the effective confinement size. Simultaneously, the tunnel barrier between the drain electrode and CQD (shown as a black arrow in Supplementary Fig. S4e) becomes thicker, as schematically shown in Supplementary Fig. S4e, which results in a decreased conductivity at the dashed line A'B' compared to the solid line AB. This is consistent with the experimental results in Supplementary Fig. S4c.
