# Supplementary material for: Single PbS colloidal quantum dot transistors
Source: Nat Commun. 2023 Nov 18;14:7486. doi: 10.1038/s41467-023-43343-7 (PMC10657373; doi:10.1038/s41467-023-43343-7)
Supplement: Supplementary file 2 — Peer Review File [file 41467_2023_43343_MOESM2_ESM.pdf]

nature portfolio

Peer Review File

Single PbS colloidal quantum dot transistors

Open Access This file is licensed under a Creative Commons Attribution 4.0 International License, which permits use, sharing, adaptation, distribution and reproduction in any medium or format, as long as you give appropriate credit to the original author(s) and the source, provide a link to the Creative Commons license, and indicate if changes were made. In the cases where the authors are anonymous, such as is the case for the reports of anonymous peer reviewers, author attribution should be to 'Anonymous Referee' followed by a clear attribution to the source work. The images or other third party material in this file are included in the article's Creative Commons license, unless indicated otherwise in a credit line to the material. If material is not included in the article's Creative Commons license and your intended use is not permitted by statutory regulation or exceeds the permitted use, you will need to obtain permission directly from the copyright holder. To view a copy of this license, visit http://creativecommons.org/licenses/by/4.0/.

REVIEWER COMMENTS

Reviewer #1 (Remarks to the Author):

The authors report the carrier transport characteristics of single PbS colloidal quantum dots (CQDs) in single-electron transistor (SET) geometries. They observed several physical properties that are specific to charge transport through nanoscale materials. Particularly the large charging energy (~150 meV) is promising to realize room temperature SETs. The QD-specific properties presented in this manuscript are intriguing, and their explanations are plausible. However, similar findings have been reported previously, though not in exactly the same system (CQD-SETs). The authors should state what is distinctive in CQD-SETs to enhance the significance of this work. Although the authors suggest the room temperature SET, they only show the data at 4 K. Additionally, the paper may suffer from some unclear results that fail to adequately support their discussions. The reviewer consider that the paper in the present form does not meet the high standard of Nature Communications. The following points should also be addressed and discussed.

1. How do the authors rule out the possibility of multi-dot SETs, considering the presence of many CQDs between the nanogap electrodes (Figure S5)?

2. Related to the previous point, some Coulomb diamonds appear unclear, such as in Figs. 2c and S1b. The shape of Coulomb diamond seems to be biased by the guidance of the broken lines. How did the authors reasonably determine the shape of the Coulomb diamond? This aspect is critical to for quantitative discussions, such as the charging energy and the energy level separation.

3. The authors attribute the Vg/Vsd-dependent excited state level to electron confinement within the CQDs (p.7-8). However, it is unclear whether such a significant effect (~50 meV, Fig. S2c) is solely induced by applied voltages. Furthermore, the tunneling conductance appears to be sensitive to electron confinement, as observed in Fig. 3c. The authors should discuss the confinement effect more quantitatively, including its impact on tunneling conductance.

4. The excited state exhibits a double split, as seen in Fig. S2a (e.g., 1, 2). Is this consistent with the electronic states formed in PbS-CQDs?

5. The CQD, acting as a Coulomb island, is surrounded by other CQDs and electrodes (Figure S5). This configuration lowers the charging energy of the Coulomb island. Surprisingly, the experimentally

measured charging energy is comparable to that of an isolated metal sphere (Fig. 3b). This observation highlights the potential advantage of employing CQDs as Coulomb islands in room-temperature SETs. The reviewer encourages the authors to delve into a more sophisticated modeling of the CQDs to elucidate the origin of this substantial charging energy.

6. The temperature dependence of the SET characteristics is essential for demonstrating the potential of CQD-SET as a room temperature device. It is necessary to present the SET characteristics at higher temperatures.

Reviewer #2 (Remarks to the Author):

In the considered paper, the Shibata et al. report the successful fabrication and characterization of single quantum dot transistors based on colloidal PbS nanocrystals. These nanocrystals have been subjects to plenty of studies regarding their electronic or optical properties, but almost all works (as correctly noted by the authors) use assemblies to understand the fundamental properties of these quantum objects. Several works appeared recently on single quantum dot optical spectroscopy, although mostly using other materials. The electronic properties on the single quantum object level have rarely been studied, and these previous results are not conclusive. This work completes the picture.

The reported results confirm the atom-like, quantized nature of the QD electronic structure, the validity of the metal sphere model for charging energy estimation, and shed light to the degree of wave function spread at higher order excited states and external electric fields. They show Kondo-effect and rationalize that it may contribute significantly to the electrical properties even at room temperatures. The work is original, and the results are robust. The interpretation is somewhat more qualitative that quantitative, but perfectly suitable for the purpose. The paper is well organized and written, the key messages are clearly laid out. I suggest publication with the correction of the following minor issues:

The title is missing an “A” from the beginning or a “-s” from the end (same for first subtitle of the Results section).

The details are missing how the air exposure was avoided (way of sample handling, encapsulation, equipment), especially since these measurements likely involved wire bonding or similar contacting typically done in air.

Figure 4 axis labels and tick labels are inconsistent in size and font.

What does the number of electrons mean in this situation? When “different number of electrons” is written, does it mean the device is testing different quantized states? Or are there charged defects that change the entire electronic structure?

The text could benefit from careful copyediting for fluency (there are several occurrences where the content is correct, but the wording is not along what is typically used in the literature, making it slightly harder to read).

Response Letter to reviewers

First, we thank the reviewers for their constructive comments on our manuscript, which indeed helped us improve the scientific quality of the manuscript. Following the reviewers' comments/suggestions, we have carefully addressed all the points mentioned by the reviewers and modified our manuscript throughout. We believe that we have properly responded to all the comments of the reviewers in this response letter and that our manuscript has been sufficiently improved for publication in Nature Communications.

The following are the point-by-point replies to the reviewers' comments:

Comment of Reviewer #1

The authors report the carrier transport characteristics of single PbS colloidal quantum dots (CQDs) in single-electron transistor (SET) geometries. They observed several physical properties that are specific to charge transport through nanoscale materials. Particularly the large charging energy (~150 meV) is promising to realize room temperature SETs. The QD-specific properties presented in this manuscript are intriguing, and their explanations are plausible. However, similar findings have been reported previously, though not in exactly the same system (CQD-SETs). The authors should state what is distinctive in CQD-SETs to enhance the significance of this work.

Our reply:

We wish to express our gratitude to the reviewer for their careful reading and valuable comments. The reviewer noted that similar results have been reported in single-electron transistors (SET) using InAs, GaAs, and Si-based quantum dots with typical sizes of ~50-100 nm [6-19]. However, in this work, we conducted experiments on SETs using colloidal quantum dots (CQDs) as Coulomb islands, and we present the following features and significance:

1. By using small CQDs with sizes less than ~5 nm, single PbS CQD transistors operate as SETs even at room temperature, as shown in Fig. R1. Moreover, due to the difficulty in fabricating SETs with single CQDs as Coulomb islands, there have been very few reports on SETs using CQDs. In this study, we present the first room-temperature SET operation in semiconductor nanocrystal systems.
2. QDs can be treated by liquid processes and enable the fabrication of artificial crystals and their utilization in various devices by selecting suitable ligands. Especially in this study, we used commercially available high-quality CQDs with oleic acid as a ligand for SETs for the first time. Oleic acid is a long-chain insulating ligand that prevents interaction with surrounding electrodes/CQDs, leading to isolated CQDs. Large charging energies in isolated CQDs play an important role in the room temperature operation of the device in this study.

3. While CQDs have been actively studied for their favorable optical properties and potential applications in optoelectronic devices, the lack of understanding of their electrical conduction characteristics has been the bottleneck for their device applications. The fundamental information on electrical transport properties at the single CQD level that we provide will help fill this gap.
4. Concerning the previous point, SETs using optically active single CQDs will lead to the realization of quantum information devices capable of both electrical and optical control of quantum states.

Following the reviewer's comment, we made significant revisions to the abstract, introduction, and conclusion sections to emphasize these distinctive points of this study on CQDs.

Although the authors suggest the room temperature SET, they only show the data at 4 K. Additionally, the paper may suffer from some unclear results that fail to adequately support their discussions. The reviewer consider that the paper in the present form does not meet the high standard of Nature Communications. The following points should also be addressed and discussed.

Our reply:

After receiving comments from the reviewer, we measured the transport characteristics at room temperature. Figure R1 shows the transport characteristics of three different single CQD transistors (with CQD diameter $d \sim 4.8$ nm) measured at room temperature. While there is a tendency for the noise to increase at higher temperatures, we still obtained diamond-like Coulomb stability diagrams for all devices, confirming that the devices operate as SETs even at room temperature. We have added these results (Fig. R1) to the revised manuscript and the Supplementary Information. To date, various SET devices such as Si-based SET structures [56-59], carbon nanotubes [60], graphene quantum dots [61], and metal nanoparticles [62] have been demonstrated to operate at room temperature. This is the first demonstration of room-temperature SET operation in semiconductor nanocrystal systems.

The reviewer mentioned some points about unclear results. They are most likely related to the results observed from samples with $d \sim 8.7$ nm (Figs. 2c and Supplementary Fig. S1b). We provide a detailed explanation of these results later in this rebuttal and in our responses to other comments. We believe that with these revisions, the paper meets the high standards of Nature Communications.

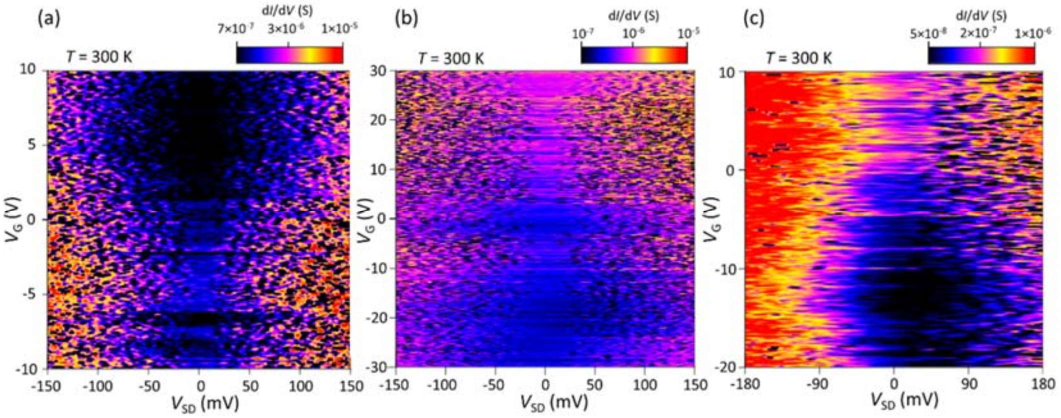

Figure R1: Coulomb stability diagrams of three different samples (a) F, (b) G, and (c) H with *d* ~ 4.8 nm measured at room temperature *T* = 300 K.

1. How do the authors rule out the possibility of multi-dot SETs, considering the presence of many CQDs between the nanogap electrodes (Figure S5)?

Our reply:

We measured numerous samples, including those exhibiting multi-dot-like transport characteristics. However, the results presented in this paper focus only on evaluating the samples whose characteristics can be interpreted as electron transport through single CQDs.

Nevertheless, as the reviewer pointed out, many surrounding CQDs may exist in some of our samples (e.g., previous Supplementary Fig. S5) and affect the electron transport by causing an increase in noise through Coulomb interactions. This is probably the case for the presented sample with a diameter *d* ~ 8.7 nm (shown in Figs. 2c and Supplementary Fig. S1b). The Coulomb diamond signatures appear particularly unclear, possibly due to the multi-dot nature. By observing the structure of the particular devices using SEM, we found that the gap size of the nanogap electrode in both samples was approximately 10 nm. Considering the dot size of *d* ~ 8.7 nm (~12 nm including oleic acid ligand), parallel quantum dot conduction is more likely to be detected than serial quantum dot conduction in this situation. In the case of parallel QD arrangements, we would have observed overlaps of two types of Coulomb diamonds. However, such behavior was not clearly observed in Fig. 2c and Supplementary Fig. S1b. Therefore, we think that the unclear Coulomb diamonds are likely due to the significant charge fluctuations in the surrounding CQDs.

We have added the related notes in the revised manuscript.

2. Related to the previous point, some Coulomb diamonds appear unclear, such as in Figs. 2c and S1b. The shape of Coulomb diamond seems to be biased by the guidance of the broken lines. How did the authors reasonably determine the shape of the Coulomb diamond? This aspect is critical to for quantitative discussions, such as the charging energy and the energy level separation.

Our reply:

The results shown in Fig. 2c and Supplementary Fig. S1b correspond to large PbS CQDs with $d \sim$ 8.7 nm. As mentioned in the main text, we believe these results correspond to measurements of the many-electron regime. In general, the single electron transport in the many-electron regime is known to be well described by the constant interaction model [9]. Therefore, in the analysis of Coulomb diamonds in Fig. 2c and Supplementary Fig. S1b, we arranged the lines forming the boundaries of the diamonds (two types of lines with positive and negative slopes) in such a way that they exhibited a gate-independent constant slope, allowing us to determine the size of the diamonds. While some Coulomb diamonds do not close, suggesting the possibility of multi-dot behavior, it should also be considered that in this system, the significant noise and instability of the characteristics may be influenced by the charge fluctuations in the surrounding QDs. Nevertheless, we believe that including the results of $d \sim$ 8.7 nm CQDs is meaningful as reference data, and we would like to continue presenting them. Additionally, the size dependence of the charging energy does not deviate significantly from the theoretical curve in Fig. 3b, which further supports the validity of including these data as a reference.

To address this point clearly, we have added the related notes in the revised manuscript.

3. The authors attribute the Vg/Vsd-dependent excited state level to electron confinement within the CQDs (p.7-8). However, it is unclear whether such a significant effect (~50 meV, Fig. S2c) is solely induced by applied voltages. Furthermore, the tunneling conductance appears to be sensitive to electron confinement, as observed in Fig. 3c. The authors should discuss the confinement effect more quantitatively, including its impact on tunneling conductance.

Our reply:

There was a mistake in part of the text in the Supplementary Information. We thank the reviewer for pointing this out. In the previous Supplementary Information, we stated that the peak spacing in the previous Supplementary Fig. S2c changed by 50 mV, suggesting that $\Delta E(N)$ increases by 50 meV. However, as shown in Fig. R2a, this peak spacing reflects $\sim 2\Delta E(N)$, which means that the increase in $\Delta E(N)$ is actually half of 50 meV, i.e., 25 meV. We apologize for the error, and we have corrected the Supplementary Information accordingly.

The main reason for such a significant change in confinement size is likely to be the large negative V_{SD} applied at the dashed line A'B'. The energy scale is very large in sample A due to the small

QD size, and at the dashed line A'B', a negative voltage of 0.1-0.3 V is applied. In such a situation, a strong electric field from the nanogap metal electrodes (with an electric field intensity of approximately 0.2 MV/cm for an applied voltage of 0.2 V and a nanogap electrode gap size of 10 nm) is applied to the CQD, leading to the modulation of the effective confinement size. Simultaneously, the tunnel barrier between the drain electrode and CQD (shown as a black arrow in Fig. R2b) becomes thicker, as schematically shown in Fig. R2b, which results in decreased conductivity at the dashed line A'B' compared to the solid line AB. This is consistent with the experimental results in Supplementary Fig. S4c.

Regarding the quantitative discussion on the confinement effect, due to the complexity of the situation and the lack of information, such as quantum dot shapes and the number of electrons in the CQD, it is not possible to provide a clear answer at this time. This remains a challenge for the future. We thank the reviewer for making us aware of this important and intriguing challenge. To make the explanation more clear/convincing to readers, we have added Fig. R2 and related notes to the revised Supplementary Information.

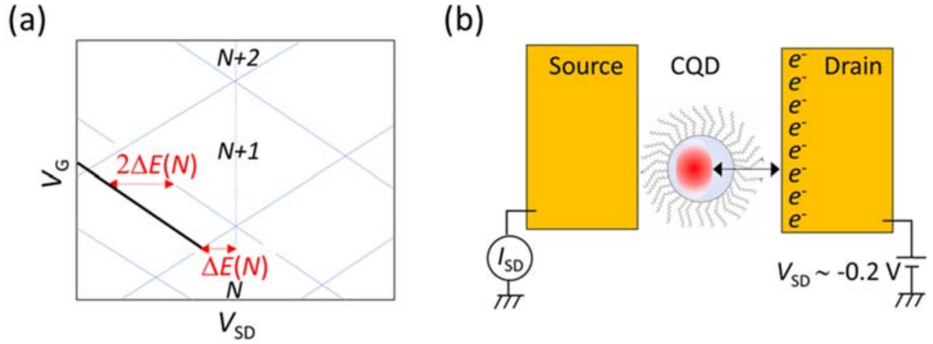

Figure R2: (a) Schematic illustration of the orbital quantization energy differences for *N* electrons, $\Delta E(N)$ derived from the excited-state line. The voltage difference between the boundary of the (N+1) Coulomb diamond and the excited-state line of the *N* electrons corresponds to ~ $2\Delta E(N)$. (b) Schematic illustrations of electron wavefunctions (shown as red) in the CQDs and nanogap source-drain electrodes under a high source-drain voltage of V_{SD} ~ 0.2 V.

4. The excited state exhibits a double split, as seen in Fig. S2a (e.g., 1, 2). Is this consistent with the electronic states formed in PbS-CQDs?

Our reply:

The origin of why the excited state appears to be split is simply due to the color scale issue. There is only one excitation level line present here. We have adjusted the color scale of this figure in the main text and supplementary information to address the misunderstanding caused by the previous color scale. We apologize for using a confusing color scale.

5. The CQD, acting as a Coulomb island, is surrounded by other CQDs and electrodes (Figure S5). This configuration lowers the charging energy of the Coulomb island. Surprisingly, the experimentally measured charging energy is comparable to that of an isolated metal sphere (Fig. 3b). This observation highlights the potential advantage of employing CQDs as Coulomb islands in room-temperature SETs. The reviewer encourages the authors to delve into a more sophisticated modeling of the CQDs to elucidate the origin of this substantial charging energy.

Our reply:

We thank the reviewer for making us aware of this important point. The decrease in the charging energy when there are metal electrodes and many CQDs in the vicinity, as noted by the reviewer, is likely due to the strong interaction between the CQD, acting as a Coulomb island, and adjacent electrodes/CQDs, allowing the wavefunctions of electrons in the CQD to spatially overlap with the adjacent electrodes/CQDs and spread. In our study, the CQDs are capped with insulating oleic acid (length ~ 2 nm) as an organic ligand, which results in weak interactions with neighboring electrodes/CQDs and minimal wavefunction overlap to the neighboring electrodes/CQDs, leading to isolated CQDs that can be explained by the isolated metal sphere model.

Furthermore, as discussed in the main text, small CQDs (samples A, B and D) are likely to be in the few-electron regime, where the spatial extension of wavefunctions is small, suggesting even weaker interactions with neighboring electrodes/CQDs, making them appear as almost isolated CQDs. However, for large CQDs where the many-electron regime is observed, the wavefunctions significantly extend within the CQD, possibly resulting in stronger interactions with neighboring electrodes/CQDs. The observed charging energy in the d ~ 8.7 nm CQD being smaller than the curve of the isolated metal sphere model could be influenced by the interactions with neighboring electrodes/CQDs. Nevertheless, there might be substantial errors in evaluating the charging energy due to the unclear Coulomb diamond in this case. Therefore, we refrain from delving further into this discussion in the main text.

Associated with the reviewer's comment, we have added related notes in the revised manuscript.

6. The temperature dependence of the SET characteristics is essential for demonstrating the potential of CQD-SET as a room temperature device. It is necessary to present the SET characteristics at higher temperatures.

Our reply:

In addition to the room temperature transport characteristics shown in Fig. R1, we also measured the temperature variation of the stability diagram, as shown in Fig. R3. The stability diagram significantly varies at different temperatures, probably due to the influence of thermal

shrinkage/expansion and/or changes in the amount of surface charge. However, we observed part of diamond-like Coulomb stability diagrams at these temperatures, suggesting that the sample indeed operates as a SET up to room temperature. It is known that the criterion for the high temperature operation of single electron transistors is $E_{add} > \sim 4k_BT$, considering thermal broadening in the single-level tunneling regime [55]. Given that E_{add} > 120 meV for CQDs with d ~ 4.5 nm, which exceeds $4k_BT$ at T = 300 K, it is reasonable to observe Coulomb diamonds even at room temperature. With increasing temperature, the conductance of the sample increases due to thermally assisted tunneling.

Associated with the reviewer's comment, we have added Fig. R3 and related notes to the revised manuscript and Supplementary Information.

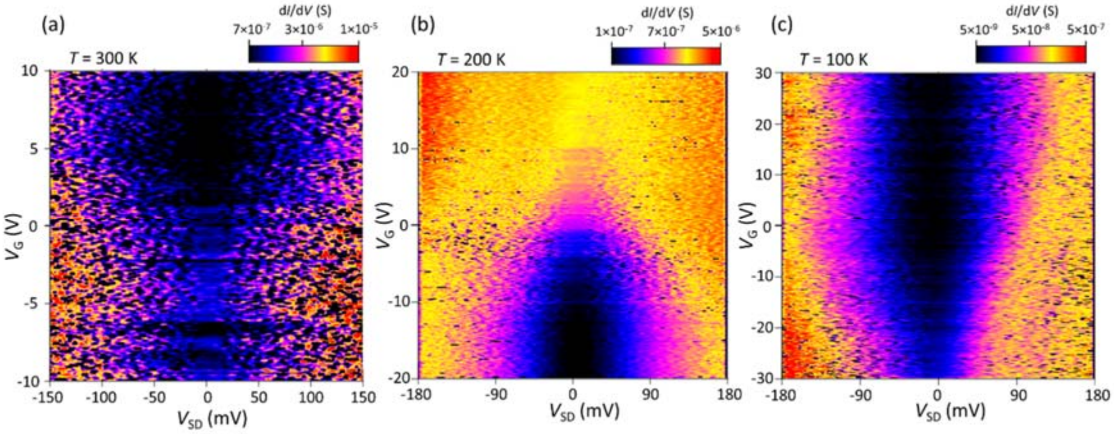

Figure R3: Coulomb stability diagrams for sample F with d ~ 4.8 nm measured at high temperatures of (a) T = 300 K, (b) 200 K, and (c) 100 K. The sample became insulating in the low V_{SD} region at T = 4 K.

Comment of Reviewer #2

In the considered paper, the Shibata et al. report the successful fabrication and characterization of single quantum dot transistors based on colloidal PbS nanocrystals. These nanocrystals have been subjects to plenty of studies regarding their electronic or optical properties, but almost all works (as correctly noted by the authors) use assemblies to understand the fundamental properties of these quantum objects. Several works appeared recently on single quantum dot optical spectroscopy, although mostly using other materials. The electronic properties on the single quantum object level have rarely been studied, and these previous results are not conclusive. This work completes the picture.

The reported results confirm the atom-like, quantized nature of the QD electronic structure, the validity of the metal sphere model for charging energy estimation, and shed light to the degree of wave function spread at higher order excited states and external electric fields. They show Kondo-effect and rationalize that it may contribute significantly to the electrical properties even at room temperatures. The work is original, and the results are robust. The interpretation is somewhat more qualitative that quantitative, but perfectly suitable for the purpose. The paper is well organized and written, the key messages are clearly laid out. I suggest publication with the correction of the following minor issues:

Our reply:

We greatly appreciate the reviewer's proper summary of our results. We are happy that the reviewer finds our work "original, and the results are robust" and recommends publishing it in Nature Communications with minor corrections.

1. The title is missing an "A" from the beginning or a "-s" from the end (same for first subtitle of the Results section).

Our reply:

We thank the reviewer for his/her feedback. We have made the necessary adjustments as suggested by the reviewer. The title has been modified to "Single PbS colloidal quantum dot transistors." Additionally, we added an "s" to the first subtitle in the Results section, making it "Single-PbS-CQD transistors."

2. The details are missing how the air exposure was avoided (way of sample handling, encapsulation, equipment), especially since these measurements likely involved wire bonding or similar contacting typically done in air.

Our reply:

The formation of thin gold nanojunctions, wire bonding, and fabrication of nanogap source-drain electrodes using the electrical break junction method were all performed in ambient air. Subsequently, we placed the devices with nanogap electrodes into a glovebox or a glove bag filled with nitrogen gas. We drop-casted diluted colloidal quantum dot solution onto the nanogap electrodes. We then dried the devices by blowing them with a nitrogen stream. We then mounted the samples in a cryostat insert under a nitrogen gas atmosphere. We introduced them into a top-loading cryostat while maintaining a nitrogen gas environment. After introducing the samples into the cryostat, we replaced the nitrogen gas with helium gas and conducted measurements in a helium gas environment.

We have added these sentences to the revised manuscript.

3. Figure 4 axis labels and tick labels are inconsistent in size and font.

Our reply:

We thank the reviewer for his/her feedback. We have made the necessary adjustments in the revised manuscript to unify the size and font of axis labels and tick labels in Fig. 4. We also made similar adjustments to the labels of other figures in the revised Supplementary Information to ensure consistency in font and size.

4. What does the number of electrons mean in this situation? When "different number of electrons" is written, does it mean the device is testing different quantized states? Or are there charged defects that change the entire electronic structure?

Our reply:

The "different number of electrons" we mentioned refers to the condition that the device is probing different quantized states. Unfortunately, in the samples measured in this study, we could not determine the exact number of electrons in the CQDs. Even though labelled with the same electron number, N, each sample may exhibit electrical conduction through different quantum states. For example, this may manifest as a significant additional energy at electron number N+1 for sample D. We do not believe that the differences in the number of electrons between the samples are significant enough to cause substantial changes to the entire electronic structure. We suspect that the differences are approximately 2, 4, or 6 electrons at most. Therefore, we do not think that there are charged defects that would alter the entire electronic structure.

We have added a related note to the revised manuscript.

5. The text could benefit from careful copyediting for fluency (there are several occurrences where the content is correct, but the wording is not along what is typically used in the literature, making it slightly harder to read).

Our reply:

We thank the reviewer for his/her feedback. We have proofread the manuscript. We tried to use more standard expressions compared to the previous version, so we believe it has become more readable.

List of major corrections

1. Following the comment of reviewer #2, the title has been modified to “Single PbS colloidal

quantum dot transistors.” Additionally, we added an “s” to the first subtitle in the Results and Discussion section, making it “Single-PbS-CQD transistors.”

2. Following the comment of reviewer #1, we made significant revisions to the abstract, introduction, and conclusion sections to emphasize the distinctive points of this study on CQDs.

3. Page 6, 1st paragraph: We added a sentence “(i.e., sample D might be probing different quantized states)” to improve the readability of this manuscript.

4. Page 6, 2nd paragraph: Following the comment of reviewer #1, we added the high-temperature transport properties (Coulomb stability diagrams) up to room temperature in the revised Fig. 3 and the revised Supplementary Figs. S2 and S3. Correspondingly, modifications were made to the main text, legend of Fig. 3, sample names, figure numbers, and reference numbers.

5. Page 10, 2nd paragraph: Following the comment of reviewer #2, we added sentences in the Methods section to provide details on how air exposure of CQDs was avoided in our study.

6. Page 12, 2nd paragraph: We added sentences explaining the effect of surrounding CQDs and electrodes on the charging energy, following the comment of reviewer #1.

7. Page 13, 1st paragraph: We added the subsection “Analysis of Coulomb stability diagrams for samples C and E ($d \sim 8.7$ nm)” and explained the possible reason for unclear Coulomb diamonds and how we determined the shape of the Coulomb diamond for CQD samples with $d \sim 8.7$ nm, following the comment of reviewer #1.

8. We added some references on the SET studies [13-20], the room-temperature operation of the SETs [55-62], and the recently published work on the CQD assembly [48], and the reference number was changed correspondingly. In addition, we have changed the reference style to meet the format of Nature Communications.

9. We have incorporated error bars in Figs. 3a and 4c. Correspondingly, we updated Fig. 3b to reflect the errors in $E_{\mathrm{add}}(n)$.

10. Supplementary information was updated following the comment of reviewer #1. In addition to Supplementary Figs. S2 and S3, we included a possible explanation for the electric field

modulation of the electron confinement size and its impact on the tunneling conductance for sample A in Supplementary Fig. S4.

REVIEWER COMMENTS

Reviewer #1 (Remarks to the Author):

The authors have extensively revised the manuscript in line with the reviewer's comments. In particular, the reviewer acknowledges that they have indeed demonstrated the room temperature operation of CQD-SETs. The overall quality of this paper has improved significantly. I have only a few minor concerns.

1. Regarding the charging energy of CQDs, which is comparable to that of an isolated metal sphere, my previous point was related to the screening effect of the electric field due to the surroundings. In simple electrostatics, the actual capacitance should always be larger than the self-capacitance ($4\pi\varepsilon r$) due to the screening effect. Such screening effects that reduce the charging energy have often been observed, for example, in gold nanoparticle-SETs. I agree that in the few-electron regime, the spatial extension of wavefunctions is small, resulting in a reduced effective size of CQDs as a Coulomb island, smaller than the geometrical size estimated from the SEM images (as illustrated in Fig. 3e). I am concerned that the agreement with the isolated metal sphere might be misleading since the Coulomb island is at least capacitively coupled with the source, drain, and gate electrodes. It would be worthwhile to compare with the self-capacitance as a reference, but the authors may also consider commenting on this point regarding the screening effect and the reduction of the effective size, in addition to discussing the agreement with the isolated metal sphere.

2. Regarding the second point in the authors' reply, I agree that the constant interaction model describes the single-electron transport in the many-electron regime. However, the boundaries of the Coulomb diamonds in Fig. 2c and Fig. s1b are ambiguous to me. Some of the dashed lines appear to represent boundaries, while others do not. Nevertheless, even without precise evaluation, considering the size of the Coulomb diamond, I agree that the charging energy of Samples C and E is likely smaller than that of the other devices. The authors may want to reconsider whether specifying the complete shape of the Coulomb diamond for Samples C and E is necessary, and they may consider removing some of the dashed lines from Fig. 2c and s1b if they are ambiguous. Alternatively, they could clarify the definition of the boundary.

Reviewer #2 (Remarks to the Author):

In the revision, the authors address all comments from both reviewers, and the noteworthiness of the work significantly increased by including the room temperature data and the experimental details.

Response letter to reviewers

First, we thank the reviewers for their comments on our manuscript. Following the reviewers' comments/suggestions, we have carefully addressed all the points mentioned by the reviewers and modified our manuscript.

The following are the point-by-point replies to the reviewers' comments:

Comment of Reviewer #1

The authors have extensively revised the manuscript in line with the reviewer's comments. In particular, the reviewer acknowledges that they have indeed demonstrated the room temperature operation of CQD-SETs. The overall quality of this paper has improved significantly. I have only a few minor concerns.

Our reply:

We wish to thank the reviewer for his or her careful reading and valuable comments. We are truly happy to hear that the reviewer finds that our manuscript "*has improved significantly.*"

1. Regarding the charging energy of CQDs, which is comparable to that of an isolated metal sphere, my previous point was related to the screening effect of the electric field due to the surroundings. In simple electrostatics, the actual capacitance should always be larger than the self-capacitance (4πεr) due to the screening effect. Such screening effects that reduce the charging energy have often been observed, for example, in gold nanoparticle-SETs. I agree that in the few-electron regime, the spatial extension of wavefunctions is small, resulting in a reduced effective size of CQDs as a Coulomb island, smaller than the geometrical size estimated from the SEM images (as illustrated in Fig. 3e). I am concerned that the agreement with the isolated metal sphere might be misleading since the Coulomb island is at least capacitively coupled with the source, drain, and gate electrodes. It would be worthwhile to compare with the self-capacitance as a reference, but the authors may also consider commenting on this point regarding the screening effect and the reduction of the effective size, in addition to discussing the agreement with the isolated metal sphere.

Our reply:

We wish to express our gratitude to the reviewer for his or her valuable comments. We agree with the reviewer's comments and added the following sentences to the main text.

"In our samples, the CQD, acting as a Coulomb island, is surrounded by other CQDs and electrodes, as shown in Fig. 1b and Supplementary Fig. S7. In this configuration, the actual capacitance should always be larger than the self-capacitance of a metal sphere quantum dot

because the Coulomb island is capacitively coupled with the electrodes and the surrounding CQDs, leading to screening of the electric field [70]. This could result in a charging energy smaller than that of the metal sphere model. However, in the few-electron regime of small CQD samples, the spatial extension of electron wavefunctions is smaller than the geometrical CQD size estimated from the SEM images, as illustrated in Fig. 3e. Therefore, the agreement between the experimentally obtained E_C in small CQDs and the metal sphere model could be the result of the compensation of both the screening effect of the surroundings and the reduced effective CQD size in the few-electron regime. In connection with this discussion, we elaborate on the contribution of ligands. In this study, the CQDs are capped with long-chain insulating oleic acid (length ~ 2 nm) as an organic ligand, which results in weak interactions (weak capacitive coupling) with neighboring electrodes/CQDs and minimal wavefunction overlap with the neighboring electrodes/CQDs, leading to more isolated CQDs that can be explained by the almost isolated metal sphere model."

In the previous revision, we added sentences addressing the potential impact of surrounding CQDs and electrodes on the charging energy from the perspective of capping ligands. This information complements the newly added discussion on the screening effect of the surroundings. Therefore, a portion of the previous response has been incorporated into the above sentences.

2. Regarding the second point in the authors' reply, I agree that the constant interaction model describes the single-electron transport in the many-electron regime. However, the boundaries of the Coulomb diamonds in Fig. 2c and Fig. s1b are ambiguous to me. Some of the dashed lines appear to represent boundaries, while others do not. Nevertheless, even without precise evaluation, considering the size of the Coulomb diamond, I agree that the charging energy of Samples C and E is likely smaller than that of the other devices. The authors may want to reconsider whether specifying the complete shape of the Coulomb diamond for Samples C and E is necessary, and they may consider removing some of the dashed lines from Fig. 2c and s1b if they are ambiguous. Alternatively, they could clarify the definition of the boundary.

Our reply:

We appreciate the reviewer's comments on our results. In accordance with the reviewer's comments, given the unclear shape of the Coulomb diamonds, we removed some of the dashed lines from unclear areas in Fig. 2c. In addition, we added the sentence "Dashed lines are drawn only in the regions where the shape of Coulomb diamonds is relatively clear" to the legend of Fig. 2. For Supplementary Fig. S1b, we removed all the dashed lines since most of the Coulomb

diamonds are ambiguous. Nevertheless, we believe that including Supplementary Fig. S1b is meaningful as reference data, and we would like to continue presenting it in the Supplementary information. We added the sentence “While dashed lines are not drawn for sample E due to the ambiguous Coulomb diamonds, the size of Coulomb diamonds in sample E would be comparable to that in sample C” to the legend of Supplementary Fig. S1. In correspondence with these changes, Figs. 3a and 3b have been updated.

Comment of Reviewer #2

In the revision, the authors address all comments from both reviewers, and the noteworthiness of the work significantly increased by including the room temperature data and the experimental details.

Our reply:

We greatly appreciate the reviewer’s careful reading and valuable comments. We are happy that the reviewer finds that “*the noteworthiness of the work significantly increased*”.

List of corrections

1. Page 12, 2nd paragraph: We added sentences explaining the effect of surrounding CQDs and electrodes on the charging energy, following the comment of reviewer #1. Correspondingly, we added reference [70].

2. Fig. 2c and Supplementary Fig. S1b: The dashed lines to determine the size of Coulomb diamonds in Fig. 2c and Supplementary Fig. S1b were removed from unclear regions. Additionally, we added the sentence “Dashed lines are drawn only in the regions where the shape of Coulomb diamonds is relatively clear” to the legend of Fig. 2. We also added the sentence “While dashed lines are not drawn for sample E due to the ambiguous Coulomb diamonds, the size of Coulomb diamonds in sample E would be comparable to that in sample C” to the legend of Supplementary Fig. S1. Correspondingly, the data in Figs. 3a and 3b and the corresponding text have been updated.

3. The manuscript has been revised to comply with the format requirements by removing the phrases "for the first time" and "extremely" in the main text. Additionally, we restricted the

number of references to 70, leading to the removal of previous references [14] and [34].

4. We have proofread the manuscript once again to enhance readability.
